# Effect Modification by Acute Coronary Syndrome Prevalence on Non-Invasive Ventilation Efficacy in Acute Cardiogenic Pulmonary Edema: A Systematic Review and Meta-Analysis of Randomized Controlled Trials

**DOI:** 10.3390/jcdd13030135

**Published:** 2026-03-12

**Authors:** Marek Tomala, Monika Durak, Magdalena Borówka, Paweł Szkarłat, Maciej Kłaczyński

**Affiliations:** 1 Center for Invasive Cardiology, Electrotherapy, and Angiology, Clinical Research Center Intercard, 30-505 Krakow, Poland; 2 Pediatric Intensive Care Unit, Department of Women, Child and Adolescent Medicine, Geneva University Hospital, 1205 Geneva, Switzerland; 3 Department of Mechanics and Vibroacoustics, Faculty of Mechanical Engineering and Robotics, AGH University of Science and Technology, 30-059 Krakow, Poland

**Keywords:** noninvasive ventilation, CPAP, BiPAP, acute cardiogenic pulmonary edema, acute coronary syndrome, myocardial infarction, meta-analysis, meta-regression, effect modification

## Abstract

Non-invasive ventilation (NIV) reduces mortality in patients with acute cardiogenic pulmonary edema (ACPE). However, the 3CPO trial reported null results. Therefore, we hypothesized that the prevalence of acute coronary syndrome (ACS) would influence the effectiveness of NIV. A systematic literature review was conducted to identify randomized controlled trials (RCTs) comparing NIV and standard oxygen therapy in patients with ACPE from PubMed, CENTRAL, and Embase databases through December 2025. Random-effects meta-analysis and REML meta-regression were utilized, and evidence quality was evaluated using GRADE. (PROSPERO: CRD420251142245). Fourteen RCTs (*n* = 1967) were included in the analysis. NIV significantly reduced hospital mortality (RR 0.75, 95% CI 0.58–0.96; I^2^ = 0%) and endotracheal intubation (RR 0.49, 95% CI 0.35–0.68). Meta-regression revealed that study-level ACS prevalence was significantly associated with the magnitude of NIV’s mortality effect (β_1_ = −0.023 per 1% increase in ACS, *p* = 0.008; R^2^ = 46.2%). The equilibrium point occurred at an ACS prevalence of 14.1% (95% CI 5.2–23.0%). At 3CPO’s ACS prevalence of 27%, the model predicted an RR of 0.75 (95% CI 0.58–0.97). The observed 3CPO RR was 0.97 (95% CI 0.66–1.43); the confidence intervals overlap substantially, and 3CPO was underpowered for mortality as an isolated endpoint. The mortality benefit of NIV appears to be associated with the prevalence of ACS among treated patients, though this ecological finding requires confirmation at the individual-patient level.

## 1. Introduction

Acute Cardiogenic Pulmonary Edema (ACPE) is a life-threatening presentation in emergency cardiology, resulting in over one million hospitalizations annually in the U.S. alone [1]. ACPE carries substantial morbidity, with in-hospital mortality rates as high as 10% and a one-year mortality rate reaching 30%, even with current medical therapy [2]. The pathophysiological process of elevated left ventricular filling pressure results in increased pulmonary capillary fluid leakage and worsening hypoxemia, necessitating ventilation in many patients with ACPE [3].

Non-invasive ventilation (NIV), in which patients receive either Continuous Positive Airway Pressure (CPAP) or Bilevel Positive Airway Pressure (BiPAP), has become a well-established method for managing ACPE. A strong physiological rationale exists for using NIV, as it reduces preload and afterload by applying positive intrathoracic pressure. A reduction in left ventricular transmural pressure reduces myocardial oxygen demand and helps recruit alveoli, thereby improving gas exchange [4,5]. Several meta-analyses have shown that NIV can reduce mortality by approximately 35–40% and the rate of endotracheal intubation by approximately 50% compared with traditional care [6,7,8,9].

Historically, the application of NIV in patients with acute coronary syndrome (ACS) presenting with pulmonary edema has been approached with considerable caution. These early concerns focused on the theoretical potential of positive-pressure ventilation to cause or exacerbate myocardial ischemia by elevating intrathoracic pressure, thereby reducing coronary perfusion. This cautious approach is reflected in several randomized controlled trials (RCTs) that excluded patients with acute myocardial infarction (AMI) or unstable angina, and in clinical guidelines [10,11,12] that recommended caution when using NIV in this population. The Cochrane Systematic Review by Berbenetz et al. (2019) [9] thoroughly evaluated the concerns surrounding the safety of NIV in patients with ACS-related ACPE. It demonstrated that NIV use did not increase the risk of AMI during NIV (RR = 1.03, 95% CI 0.91–1.16) [9]. These results provided reassurance against the original concerns and suggested that NIV can be safely used in patients with ACS-related ACPE.

Although there is substantial cumulative evidence for NIV effectiveness, there remains significant variability in outcomes across individual trials. The 3CPO trial has been notable in this regard, as it was the largest randomized clinical trial to date (*n* = 1069) and found no reduction in mortality among participants treated with NIV. Consequently, it contributed to uncertainty about the overall effect size of NIV [13]. When sensitivity analysis is limited to high-quality trials, the estimated reduction in mortality with NIV is not statistically significant (RR 0.81; 95% CI 0.61–1.06) [9]. This disparity between individual-trial results and meta-analysis summary results suggests that additional variables likely act as confounders or modifiers of NIV’s effect in determining which patients will benefit most.

Weng et al. were the first to systematically demonstrate that ischemic etiology modifies the effectiveness of NIV [14]. Using a meta-regression, they found that CPAP produced greater reductions in mortality among those who had developed pulmonary edema due to acute MI or ischemia; the meta-regression indicated that this greater mortality reduction was related to a 17.4% average decrease in RR (95% CI = 0.7–31.2%) per 10% increase in the proportion of patients with acute MI or ischemia.

The observed association also suggested that there is a larger numerical difference in benefit between trials with >50% of patients with myocardial infarction (RR = 0.43, 95% CI, 0.17–1.07), as opposed to trials with <10% of patients with myocardial infarction (RR = 0.92, 95% CI, 0.76–1.10), indicating that one possible explanation for the observed variability in the effectiveness of NIV treatments could be due to the different prevalence rates of ACS across trial populations [14].

A large observational cohort study by Qu et al. (2022) of 1257 patients with acute systolic heart failure after an ACS event demonstrated that was independently associated with a 34 percent reduction in one-year all-cause mortality (HR 0.674; *p* = 0.045) and had significant decreases in both hospital intubations (3.5% vs. 6.36%; *p* = 0.027) and in-hospital cardiac deaths (1.44% vs. 3.89%; *p* = 0.027) [15]. Carrillo-Alemán et al. found that AMI was the only etiology of cardiogenic shock independently associated with failure to wean from NIV. However, they noted a significant reduction in mortality among patients with stage C (SCAI classification) cardiogenic shock treated with NIV compared to those treated with invasive ventilation (10.1% vs. 32.9%, *p* < 0.00001) [16]. The above-mentioned cohort studies are hypothesis-generating and require systematic evaluations.

We hypothesized that the number of participants with Acute Coronary Syndromes (ACS) in randomized trials could serve as a primary factor moderating the NIV treatment effect, accounting for the variability between studies. If NIV benefits differ significantly by the proportion of patients with ACS in a study. This finding would have direct clinical implications, as it would suggest that patients who suffer from ACPE due to ischemic causes may derive greater benefit from NIV treatment. To test our hypothesis, we conducted a systematic review and meta-analysis of randomized controlled trials comparing NIV with usual medical treatment in ACPE, along with a prespecified meta-regression to assess whether ACS prevalence is a continuous moderator of mortality.

## 2. Materials and Methods

### 2.1. Protocol and Registration

This systematic review and meta-analysis were conducted in accordance with the Preferred Reporting Items for Systematic Reviews and Meta-Analyses (PRISMA) 2020 guidelines [17] (Supplementary Table S6) and the Cochrane Handbook for Systematic Reviews of Interventions version 6.5 [18]. The protocol was prospectively registered in the International Prospective Register of Systematic Reviews (PROSPERO: CRD420251142245) on 28 October 2025, before formal screening. The review adhered to the registered protocol with prespecified amendments documented in the PROSPERO record (see Section 2.13 for deviations from the protocol).

### 2.2. Eligibility Criteria

Studies were selected according to the PICOS framework. Participants: Adult patients (≥18 years) with acute cardiogenic pulmonary edema (ACPE) of any etiology, including acute coronary syndrome (ACS), hypertensive crisis, valvular dysfunction, or decompensated chronic heart failure. No restrictions were applied regarding sex, ethnicity, or geographic location. Intervention: Non-invasive ventilation (NIV), defined as continuous positive airway pressure (CPAP) or bilevel positive airway pressure (BiPAP), delivered via facemask or nasal interface, in addition to standard medical care. Comparator: Standard medical care (SMC) alone, including supplemental oxygen therapy (nasal cannula, Venturi mask, or non-rebreather mask) and pharmacological treatment (diuretics, vasodilators, morphine, inotropes as clinically indicated). Outcomes: The primary outcome was hospital mortality (in-hospital death), with 30-day all-cause mortality used as a surrogate when hospital mortality was unavailable. Secondary outcomes included the endotracheal intubation rate, incidence of acute myocardial infarction during treatment, treatment intolerance, intensive care unit (ICU) length of stay, and hospital length of stay. Study design: Randomized controlled trials (RCTs) and quasi-randomized trials were eligible for inclusion.

Studies were also excluded if they: (1) compared CPAP vs. BiPAP in the absence of an oxygen therapy control; (2) included patients with other causes of acute cardiogenic pulmonary edema (e.g., ARDS, pneumonia, COPD exacerbation); (3) used a different ventilatory modality (e.g., proportional assist ventilation, high-flow nasal cannula, etc.; (4) investigated timing differences between early and late use of NIV and did not include a non-NIV control; (5) did not report ACS prevalence data by study arm; or (6) were only presented at a conference and had no corresponding full-text article posted within 24 months. A multi-arm trial that compared CPAP and BiPAP with a single-oxygen-therapy control was acceptable for inclusion, with the single control split across arms to prevent double-counting per the Cochrane Handbook guidelines [18].

### 2.3. Information Sources

A comprehensive literature search was conducted across six electronic databases: MEDLINE via PubMed (1990–December 2025), Cochrane Central Register of Controlled Trials (CENTRAL, Issue 12, 2025), Embase via Ovid (1990–December 2025), CINAHL Plus via EBSCO (1990–December 2025), LILACS (1990–December 2025), and the Database of Abstracts of Reviews of Effects (DARE; archival search of records through March 2015, when the database was discontinued). Trial registries searched included ClinicalTrials.gov and the World Health Organization International Clinical Trials Registry Platform (WHO ICTRP). Reference lists of included studies, prior systematic reviews [6,7,8,9], and relevant clinical practice guidelines [10,11,12] were manually searched for additional eligible studies. Authors of studies with incomplete outcome data were contacted via email with two follow-up attempts at 14-day intervals. No language restrictions were applied; non-English publications were translated by native speakers.

### 2.4. Search Strategy

The search strategy combined Medical Subject Headings (MeSH) terms and free-text keywords for the concepts of non-invasive ventilation (“non-invasive ventilation” [MeSH], “continuous positive airway pressure” [MeSH], NIV, CPAP, BiPAP, BPAP, “bilevel positive airway pressure”, “positive pressure ventilation”) AND acute heart failure/pulmonary edema (“pulmonary edema” [MeSH], “heart failure” [MeSH], “cardiogenic pulmonary edema”, “acute heart failure”, “cardiac failure”) AND study design filters for randomized controlled trials. Boolean operators (AND, OR) were used to combine search concepts. The search was limited to human studies published from 1 January 1990 to 31 December 2025. The complete search strategy for each database is provided in Supplementary Table S5. The search was last updated on 15 December 2025.

### 2.5. Selection Process

**Figure 1:**
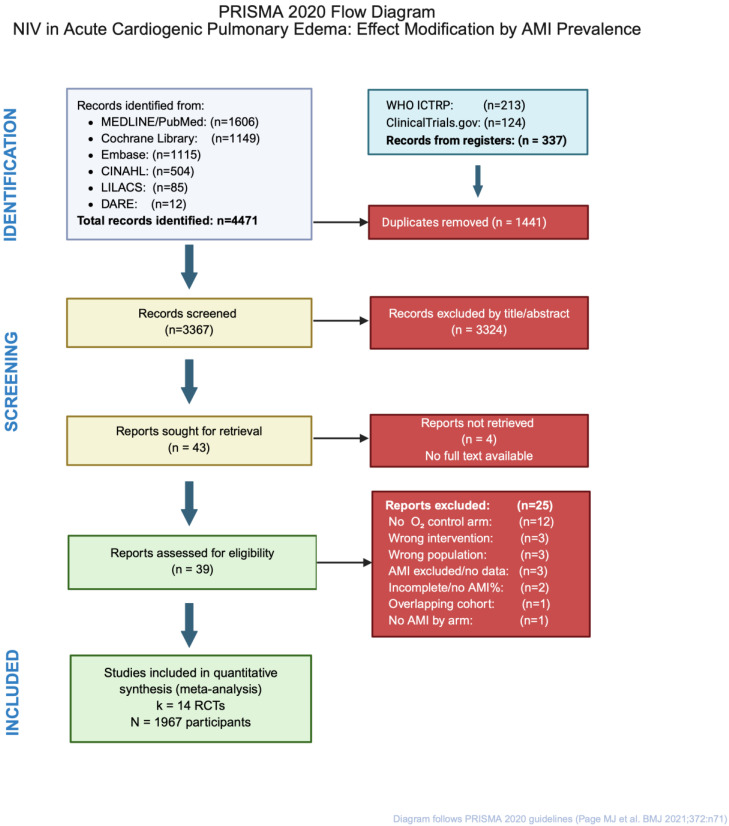
PRISMA 2020 [17] flow diagram of study selection. Records were identified from six electronic databases (PubMed, Cochrane CENTRAL, Embase, Web of Science, CINAHL, LILACS) and two trial registries (ClinicalTrials.gov, WHO ICTRP). After deduplication, titles and abstracts were screened independently by two reviewers. Full-text articles were assessed for eligibility based on predefined inclusion criteria. Fourteen randomized controlled trials met all inclusion criteria and were included in the quantitative synthesis.

Records identified from database searches were exported to Covidence systematic review software (Veritas Health Innovation, Melbourne, Australia) for deduplication. A total of 4808 records were identified; after removal of 1441 duplicates, 3367 unique records underwent title and abstract screening. Screening was performed independently by two reviewers (M.T. and M.D.) against predefined eligibility criteria. Studies deemed potentially relevant by either reviewer advanced to full-text assessment. Of the 43 reports sought for retrieval, 4 could not be obtained (Chinese-language publications and conference abstracts without full-text availability; Supplementary Table S4). The remaining 39 full-text articles were independently evaluated by the same two reviewers, with reasons for exclusion documented at this stage; 25 reports were excluded (Supplementary Table S4). Disagreements were resolved through discussion; persistent disagreements were adjudicated by a third reviewer (M.B.). Inter-rater reliability was assessed using Cohen’s kappa statistic (κ = 0.89), indicating excellent agreement. The study selection process is presented in a PRISMA 2020 flow diagram [17] (Figure 1).

### 2.6. Data Collection Process

Two independent reviewers (M.T. and M.D.) used a pilot-tested, standardized data extraction form to extract all relevant data. The following variables were collected: study information (first author, year of publication, country, setting, study design, sample size, duration of follow-up); participant demographics (mean age, sex distribution, baseline arterial pH); intervention details (NIV modality, pressure settings, interface type, duration of application); comparator details (oxygen delivery method, flow rate); outcome data (number of events and total participants in each arm for dichotomous outcomes; means and standard deviations for continuous outcomes); and the primary moderator variable, ACS prevalence.

The ACS index was defined as the proportion of patients with acute myocardial infarction (AMI) or an acute ischemic etiology, temporally and causally related to the index ACPE episode, occurring within 0–72 h of admission or randomization. This definition aligns with the Fourth Universal Definition of Myocardial Infarction criteria, used as a conceptual framework for defining the temporal window (0–72 h) and the scope of acute ischemic events, not for retrospective patient-level reclassification. Remote or prior ischemia or infarction unrelated to the index event was excluded. Where studies reported AMI and acute myocardial ischemia as separate diagnostic categories (Bersten 1991 [19], Masip 2000 [20], Park 2001 [21], Park 2004 [22]), both were summed to derive the ACS Index (Supplementary Table S7). Where available, AMI diagnoses were verified against the original investigators’ reported criteria, including symptoms, electrocardiographic changes, and elevation of cardiac biomarkers. Secondary data from high-quality reanalyses (e.g., Winck et al. [7]) were used when primary reporting was incomplete, and the use was transparently documented. Discrepancies between reviewers were resolved by consensus or third-party adjudication. ACS prevalence was calculated for the entire study population rather than separately by treatment arm, as diagnoses were established at or before enrollment (i.e., before randomization), and randomization is expected to balance baseline characteristics between arms.

### 2.7. Risk of Bias Assessment

The risk of bias was assessed independently by two reviewers (M.T. and M.D.) using the Cochrane Risk of Bias tool version 1 (RoB 1). We used RoB 1 because the included trials (1985–2011) predated RoB 2 [23]. The retrospective application of RoB 2 would be anachronistic, as it would assess historical studies against methodological standards that did not exist when these trials were designed.

Each study was evaluated across seven domains: (D1) random sequence generation (selection bias); (D2) allocation concealment (selection bias); (D3) blinding of participants and personnel (performance bias); (D4) blinding of outcome assessment (detection bias); (D5) incomplete outcome data (attrition bias); (D6) selective reporting (reporting bias); and (D7) other bias (bias due to problems not covered elsewhere, including baseline imbalances, early stopping, and funding sources). Each domain was judged as “low risk,” “unclear risk,” or “high risk” of bias according to the Cochrane Handbook criteria [18]. Given the nature of the NIV intervention, the blinding of participants and personnel (D3) was not feasible in any trial; however, this domain was not automatically rated as high risk if adequate measures were taken to minimize performance bias (e.g., standardized co-interventions, objective outcome definitions). For Domain 2 (allocation concealment), studies using envelopes that were not demonstrably opaque and sequentially numbered were classified as high risk, consistent with the Cochrane guidance that unsealed or non-opaque envelopes provide insufficient protection against selection bias. This criterion was applied to Masip et al. [20], in which allocation concealment relied on closed envelopes attached to rounding sheets—a method lacking both sealing and verified opacity.

An overall risk of bias judgment was derived for each study: “low risk” if all key domains (D1, D2, D5) were at low risk, “high risk” if any key domain was at high risk, and “unclear risk” otherwise. Four studies (*n* = 1327) were classified as having a low risk of bias, and ten studies (*n* = 640) as having a high risk of bias [24]. Disagreements were resolved through discussion with a third reviewer (M.B.). Risk of bias summary and traffic-light plots were generated using the robvis package in R. Prespecified subgroup analyses comparing trials with low versus high risk of bias were performed to assess the robustness of the pooled estimates.

### 2.8. Effect Measures and Data Synthesis

For dichotomous outcomes (mortality, intubation, and AMI incidence), risk ratios (RR) with 95% CI were calculated using the inverse-variance method. For studies with zero events in one or both arms [20,21], a continuity correction of 0.5 was applied to enable inclusion in the pooled analysis. For continuous outcomes (length of stay), mean differences (MDs) with 95% CI were calculated using the inverse-variance method. Effect sizes were pooled using a random-effects model with the DerSimonian-Laird estimator to account for anticipated between-study heterogeneity arising from differences in clinical settings, NIV protocols, and patient populations. Sensitivity analyses were performed using restricted maximum likelihood (REML) estimation and a fixed-effect model (Mantel-Haenszel) to assess the robustness of the pooled estimates.

Statistical heterogeneity was assessed using the Cochran Q test (α = 0.10 for significance) and quantified using the I^2^ statistic, which was interpreted as low (<25%), moderate (25–50%), substantial (50–75%), or high (>75%) heterogeneity [18]. The between-study variance (τ^2^) and 95% confidence intervals were estimated. When three or more studies were pooled, 95% prediction intervals were calculated to estimate the range of true effects expected in future similar studies. Forest plots were generated to display individual study effects with 95% CI, pooled estimates, and heterogeneity statistics.

### 2.9. Meta-Regression Analysis

To explore potential effect modification by ACS prevalence, a random-effects meta-regression was conducted using the restricted maximum likelihood (REML) estimator in R version 4.5.2 (R Foundation for Statistical Computing, Vienna, Austria) with the metafor package version 4.8-0. The primary model regressed the natural logarithm of the risk ratio (log RR) for hospital mortality against ACS prevalence (%) as a continuous moderator. The model parameters [25] included the intercept (β_0_), the regression coefficient (β_1_, per 1% increase in ACS prevalence), and the proportion of between-study variance explained (R^2^). The statistical significance of the moderating effect was assessed using the Wald test, with *p* < 0.05 considered significant.

The equilibrium point, defined as the ACS prevalence at which the predicted RR equals 1.00 (null effect), was calculated as−β_0_/β_1_, with 95% confidence intervals (CIs) derived using the delta method. Model fit was evaluated using the residual heterogeneity statistic (Qresidual, df = k − 2, where k is the number of studies) and the coefficient of determination (R^2^). Influential observations were identified using Cook’s distance (threshold >4/k, approximately 0.29 for 14 studies) and leverage values exceeding twice the average leverage (2p/*n*, where *p* = number of parameters, including intercept, and *n* = number of studies). Studies meeting these criteria underwent sensitivity analyses with sequential exclusions. Predicted risk ratios with 95% confidence intervals were calculated at clinically relevant ACS thresholds (10%, 14.1% [equilibrium], 20%, 27% [3CPO], 50%, and 100%). The meta-regression bubble plot displays study-level data with circle sizes proportional to the inverse-variance weights, fitted regression line, and 95% confidence bands.

### 2.10. Subgroup and Sensitivity Analyses

Prespecified subgroup analyses were conducted for hospital mortality in relation to: (1) the percentage of patients with Acute Coronary Syndrome (ACS), where ≥25% versus <25% was used as the cut-off, approximating the weighted mean ACS prevalence across included studies (27.1%) (2) the level of risk of bias (high vs. low); (3) NIV modality (CPAP only vs. BiPAP or mixed); and (4) clinical setting (Emergency Department/Prehospital vs. ICU/CCU).

Between-subgroup differences were tested using the Q-test for subgroup heterogeneity; *p* < 0.10 indicated potential effect modification. Prespecified sensitivity analyses included (1) exclusion of the largest trial (*n* = 1069) to assess its influence on pooled estimates [13]; (2) exclusion of studies with zero events in either arm [20,21]; (3) comparison of fixed-effect and random-effects models; and (4) comparison of DerSimonian-Laird and REML variance estimators. Leave-one-out analyses were performed by sequentially removing each study to identify the influential studies (Supplementary Table S1, Figure S1).

### 2.11. Publication Bias Assessment

Publication bias was assessed visually using contour-enhanced funnel plots of effect size (log RR) against standard error, and statistically using Egger’s weighted regression test for funnel plot asymmetry (α = 0.10) [26]. The Duval and Tweedie trim-and-fill methods were applied to estimate the number of potentially missing studies due to publication bias and to calculate an adjusted pooled effect that accounts for asymmetry [27]. Published reports were compared with trial registrations (ClinicalTrials.gov, WHO ICTRP) and, where available, with study protocols to identify selective outcome-reporting bias. The potential impact of publication bias was incorporated into the GRADE certainty assessments.

### 2.12. Certainty of Evidence Assessment

The certainty of the evidence for each outcome was assessed using the Grading of Recommendations Assessment, Development, and Evaluation (GRADE) framework [28]. Evidence from RCTs was initially rated as high certainty and evaluated across five domains: risk of bias, inconsistency, indirectness, imprecision, and publication bias. The certainty of evidence was downgraded by one or two levels per domain and rated as high, moderate, low, or very low. For imprecision, the optimal information size (OIS) was calculated assuming a control event rate of 14.1% (derived from pooled control arm data), a relative risk reduction of 25%, α = 0.05 (two-sided), and power (1 − β) = 0.80, yielding an OIS of approximately 2700 participants. The total sample size of 1967 participants did not meet this threshold; however, as the 95% confidence interval for mortality excluded the null (RR = 1.0), imprecision was not downgraded. Upgrading for a large magnitude of effect (RR < 0.5 or >2.0 with narrow CI), dose–response gradient, or plausible residual confounding was considered where applicable.

### 2.13. Deviations from Registered Protocol

The following deviations from the registered PROSPERO protocol (CRD420251142245) occurred and were documented prospectively: (1) Scope modification: The original protocol focused on NIV in ST- elevation myocardial infarction undergoing primary percutaneous coronary intervention (PCI). Following preliminary searches identifying only three PCI-specific studies with insufficient statistical power for quantitative synthesis, the scope was expanded before formal screening to include all RCTs of NIV in ACPE with extractable ACS prevalence data, enabling meta-regression analysis of ACS prevalence as a continuous effect modifier. This amendment was documented in PROSPERO before data extraction. (2) Database modifications: LILACS (*n* = 85 records) and DARE (*n* = 12 records; archival search through March 2015) were added post-hoc to capture the Latin American literature and existing systematic reviews. Web of Science (SCI) and Scopus, listed in the original protocol, were not searched; the yield from six databases (*n* = 4471) and two trial registries (*n* = 337) was deemed sufficient for comprehensive coverage, as all 14 included RCTs were indexed in MEDLINE and/or the CENTRAL of Controlled Trials. (3) risk of bias tool: The protocol listed both Cochrane RoB-1 and RoB-2. Given that all included trials were conducted between 1985 and 2011, only the original RoB 1 tool was applied, as it reflects the methodological standards and reporting conventions of that era; application of RoB 2—designed for prospective assessment of contemporary trials—would have been methodologically inappropriate for this historical evidence base. These deviations are unlikely to have affected the completeness or validity of the review findings.

### 2.14. Software and Data Availability

All statistical analyses were performed using R version 4.5.2 (R Foundation for Statistical Computing, Vienna, Austria) with the following packages: metafor version 4.8-0 for meta-analysis and meta-regression [25], robvis for risk-of-bias visualization [24], and ggplot2 for graphical outputs. Forest plots and funnel plots were generated using the forest () and funnel () functions in metafor. The complete analytical code, extracted dataset, and study-level data are available in the Supplementary Materials. Screening and study selection were conducted using Covidence version (Veritas Health Innovation, Melbourne, Australia). GRADE evidence certainty assessments were performed using GRADEpro GDT (McMaster University, Hamilton, ON, Canada) [29].

## 3. Results

### 3.1. Study Selection

The systematic search identified 4808 records across six databases and two trial registries (Figure 1). After removing 1441 duplicates, 3367 unique records underwent title and abstract screening; 3324 were excluded for failing to meet eligibility criteria. Of the 43 full-text reports sought, 4 could not be obtained (Chinese-language publications and conference abstracts without full text). The remaining 39 reports were assessed for eligibility; 25 were excluded with reasons documented in Supplementary Table S4. The most common reasons for exclusion were: comparison of CPAP versus BiPAP without standard oxygen control (*n* = 8), lack of extractable ACS prevalence data (*n* = 6), non-randomized design (*n* = 5), and timing comparison studies without a non-NIV control (*n* = 4). Notably, Plaisance [30] was excluded because it compared immediate versus delayed CPAP initiation rather than NIV versus standard care. In [31], CPAP was compared with BiPAP without an oxygen-control arm; [32] did not report AMI as a safety outcome; and [33] did not report ACS prevalence data. Fourteen randomized controlled trials [13,19,20,21,22,34,35,36,37,38,39,40,41,42], comprising 1967 participants, met all inclusion criteria and were included in the quantitative synthesis.

### 3.2. Study Characteristics

Characteristics of the 14 included trials are presented in Table 1.

Individual study sample sizes ranged from 22 to 1069 participants (median: 50; IQR: 37–89), spanning 26 years (1985–2011). The largest trial (Gray et al., 2008; 3CPO) [13] accounted for 54.4% of the total sample. The emergency department and prehospital settings accounted for 1699 participants (86.4%), while the intensive care unit and coronary care unit settings accounted for 268 participants (13.6%). Seven studies examined CPAP alone (527 participants), while seven studies examined BiPAP alone or included both CPAP and BiPAP as treatment options (1440 participants). All studies used facemask interfaces; two early studies [38,39] additionally permitted nasal mask use.

The weighted mean age across studies was 77.1 years (range: 64–84 years), and 46.1% of participants were male. Baseline arterial pH was reported in 10 studies, ranging from 7.17 to 7.35, indicating moderate-to-severe respiratory acidosis. The acute coronary syndrome prevalence (ACS index) ranged from 14.6% [35] to 100% [39], with a weighted mean of 27.1% and a median of 31.2%. Five studies reported ACS prevalence ≤ 25% (*n* = 564), and nine reported ACS prevalence > 25% (*n* = 1403).

### 3.3. Risk of Bias Assessment

Risk of bias assessment using the Cochrane RoB 1 tool [18] is summarized in Supplementary Figure S4 (traffic-light plot) and Supplementary Figure S2 (summary plot). Four studies representing 1327 participants (67.5% of the total sample) were classified as low overall risk of bias: [13,19,34,35]. These studies employed adequate random sequence generation (computer-generated lists or central telephone randomization) and robust allocation concealment (sealed opaque envelopes or central allocation). Ten studies (*n* = 640, 32.5%) were classified as high overall risk primarily due to inadequate or unclear allocation concealment.

At the domain level, random sequence generation (D1) was adequate in 10 of 14 studies; four studies used methods with unclear adequacy (sealed envelopes without specification of opacity or sequential numbering). Allocation concealment (D2) was the most problematic domain: only four studies demonstrated adequate concealment [20], which was classified as high risk because envelopes attached to rounding sheets were used rather than sealed opaque envelopes. Blinding of participants and personnel (D3) was not feasible in any study, given the nature of the NIV intervention; however, all studies used objective outcome definitions (death, intubation) that minimized the impact of performance bias. Incomplete outcome data (D5) were low risk in 13 studies, with intention-to-treat analysis and minimal loss to follow-up (<5%). Selective reporting (D6) could not be assessed for most studies because they lacked pre-registered protocols; however, all studies reported the primary outcomes specified in their methods sections.

### 3.4. Primary Outcome: Hospital Mortality

**Figure 2:**
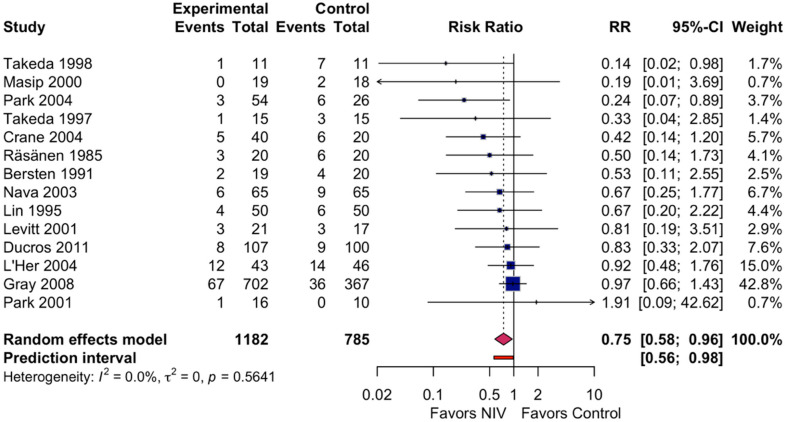
Forest plot of hospital mortality comparing non-invasive ventilation versus standard oxygen therapy in patients with acute cardiogenic pulmonary edema [13,19,20,21,22,34,35,36,37,38,39,40,41,42]. Squares represent the risk ratio for each study, with the square size proportional to the study’s weight. Horizontal lines indicate 95% confidence intervals. The diamond represents the pooled risk ratio with its 95% confidence interval. The solid vertical line indicates no effect (RR = 1.0). RR, risk ratio; CI, confidence interval; NIV, non-invasive ventilation.

All 14 studies reported hospital mortality data, encompassing 1967 participants (Figure 2).

In the NIV group, 116 of 1182 patients (9.8%) died compared with 111 of 785 patients (14.1%) in the control group. Two studies had zero events in one treatment arm: [20] (NIV 0/19, control 2/18) and [21] (NIV 1/16, control 0/10); these were included using a continuity correction of 0.5.

Random-effects meta-analysis demonstrated that NIV significantly reduced hospital mortality compared with standard oxygen therapy (RR 0.75, 95% CI 0.58–0.96; *p* = 0.022). Statistical heterogeneity was absent (I^2^ = 0%; Cochran Q = 11.57 (df = 13, *p* = 0.56; τ^2^ = 0). The 95% prediction interval (0.56–0.98) indicated that, in a similar new clinical trial, the actual treatment effect for NIV is expected to be beneficial or neutral. In terms of raw numbers, 35 fewer deaths are expected per 1000 patients treated with NIV (95% CI 6–59 fewer deaths), corresponding to an estimated number needed to treat (NNT) of 29 (95% CI 17–167). Both fixed-effect (RR 0.75, 95% CI 0.58–0.96) and REML sensitivity analyses (RR 0.75, 95% CI 0.58–0.97) yielded consistent results, as expected given the absence of between-study heterogeneity (τ^2^ = 0). Q = 11.57 (df = 13, *p* = 0.56).

### 3.5. Secondary Outcome: Endotracheal Intubation

**Figure 3:**
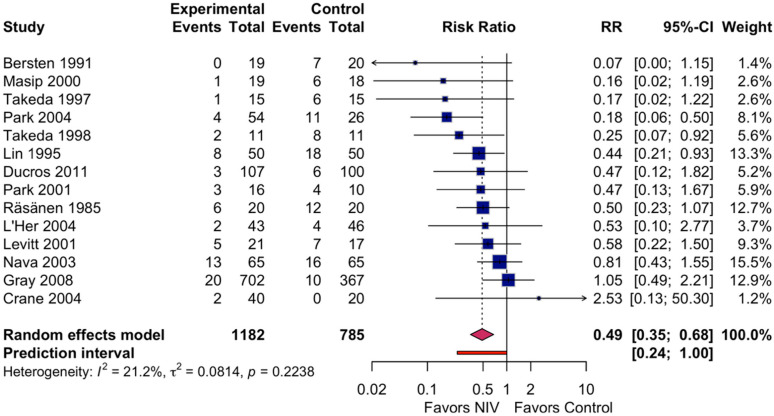
Forest plot of endotracheal intubation comparing non-invasive ventilation versus standard oxygen therapy in patients with acute cardiogenic pulmonary edema [13,19,20,21,22,34,35,36,37,38,39,40,41,42]. Squares represent the risk ratio for each study, with the square size proportional to the study’s weight. Horizontal lines indicate 95% confidence intervals. The diamond represents the pooled risk ratio with its 95% confidence interval. The solid vertical line indicates no effect (RR = 1.0). RR, risk ratio; CI, confidence interval; NIV, non-invasive ventilation.

All 14 studies reported endotracheal intubation rates, with a total of 1967 participants (Figure 3).

In the NIV group, 70 of 1182 patients (5.9%) required intubation compared with 115 of 785 patients (14.6%) in the control group.

Random-effects meta-analysis demonstrated that NIV significantly reduced the need for endotracheal intubation (RR 0.49, 95% CI 0.35–0.68; *p* < 0.001). Heterogeneity was low (I^2^ = 21%; Cochran Q = 16.5, df = 13, *p* = 0.22; τ^2^ = 0.08). In absolute terms, NIV was associated with 76 fewer intubations per 1000 patients treated (95% CI 47–96 fewer), corresponding to an NNT of 13 (95% CI 10–21).

The GRADE certainty of evidence for intubation was rated as moderate, downgraded by 1 level for risk of bias due to the impossibility of blinding the intervention and the subjective nature of intubation decisions (Table 2).

### 3.6. Meta-Regression: Effect Modification by ACS Prevalence

**Figure 4:**
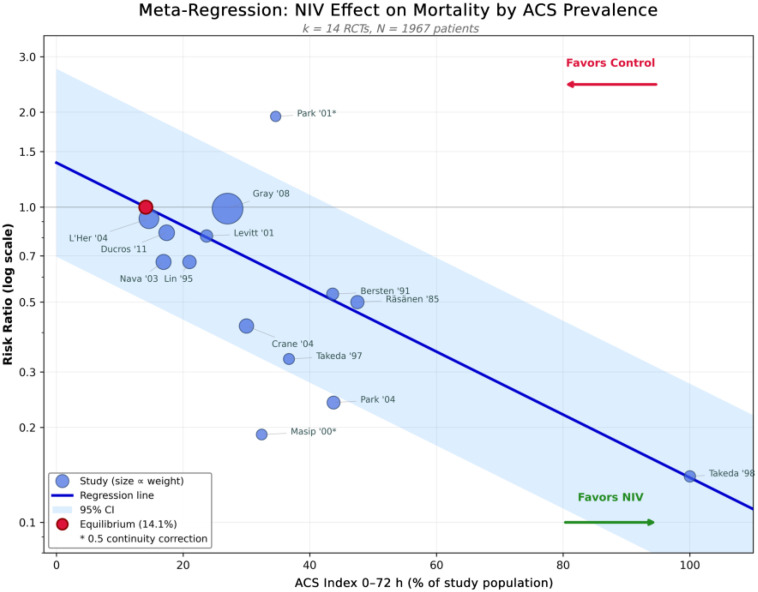
Meta-regression bubble plot showing the association between acute coronary syndrome (ACS) prevalence and the effect of non-invasive ventilation on hospital mortality. Each circle represents an individual study, with the circle size proportional to the study’s weight. The regression line (solid) with a 95% confidence interval (shaded area) shows a decreasing risk ratio (greater NIV benefit) with increasing ACS prevalence. The horizontal dashed line indicates no effect (RR = 1.0). The equilibrium point (14.1% ACS) represents the threshold below which NIV shows no mortality benefit. Studies marked with an asterisk (*) required a 0.5 continuity correction for zero events: Masip et al., 2000, [20], and Park et al., 2001, [21]. The regression coefficient for the dose–response was β_1_ = −0.023 (Standard Error = 0.007) for every 1% increase in ACS prevalence (*p* = 0.008). This indicates that each 10-percentage-point increase in ACS prevalence corresponds to approximately a 21% greater reduction in mortality with NIV (Relative Risk = 0.79).

Random-effects meta-regression analysis revealed a statistically significant association between ACS prevalence and the magnitude of NIV benefit on hospital mortality (Figure 4).

The model accounted for 46.2% of the variation among studies (R-squared = 0.462), reducing the unexplained variability from Q = 11.57 (df = 13, *p* = 0.56) to Q residual = 4.71 (df = 12, *p* = 0.94).

The “equilibrium” point—the ACS prevalence level at which the predicted effect of NIV is equivalent to no effect (relative risk 1.00)—was estimated to be at 14.1% (95% confidence interval 5.2–23.0%). At levels below this threshold, NIV had no advantage over standard oxygen therapy for decreasing mortality. At ACS prevalence levels above this threshold, NIV was associated with progressively greater reductions in mortality.

Predicted risk ratios at clinically relevant ACS prevalence thresholds were:

- ACS prevalence 10%: RR 1.10 (95% CI 0.72–1.67).
- ACS prevalence 20%: RR 0.87 (95% CI 0.66–1.15).
- ACS prevalence 27% (corresponding to the 3CPO trial [13]): RR 0.75 (95% CI 0.58–0.97).
- ACS prevalence 50%: RR 0.46 (95% CI 0.26–0.82).
- ACS prevalence 100%: RR 0.15 (95% CI 0.04–0.52).

The extreme ACS prevalence values (especially 100%) are purely mathematical extrapolations based on a log-linear model and cannot be used to make clinical predictions. The dose–response relationship is best understood by examining the actual data range (i.e., approximately 15–50% ACS prevalence). Additional complete predicted risk ratios at additional thresholds are listed in Table S8.

Influence diagnostics identified [21] (Cook’s distance = 0.31, >0.29) and [39] (Leverage = 0.32, >2*p/*n* = 0.29) as potential influential cases. When we excluded these two studies from the dataset, we obtained very similar estimates (β_1_ = −0.021, *p* = 0.012; Equilibrium Point = 15.3%). This confirmed that the dose–response relationship was robust.

### 3.7. Subgroup Analyses

Predefined subgroup analyses for hospital mortality are presented in Supplementary Table S3.

In the ACS prevalence subgroup analysis, trials with ACS > 25% (9 studies, *n* = 1403) showed a significant mortality benefit (RR 0.65, 95% CI 0.47–0.91; I^2^ = 0%), whereas trials with ACS ≤ 25% (5 studies, *n* = 564) did not (RR 0.93, 95% CI 0.61–1.43; I^2^ = 0%). The test for subgroup differences approached significance (Q = 2.89, df = 1, *p* = 0.089), providing supportive evidence consistent with the meta-regression findings.

In the risk of bias subgroup analysis, low-risk-of-bias studies (4 studies, *n* = 1327) did not demonstrate a significant mortality benefit (RR 0.88, 95% CI 0.64–1.22; I^2^ = 0%), whereas high-risk-of-bias studies (10 studies, *n* = 640) showed a larger benefit (RR 0.54, 95% CI 0.35–0.83; I^2^ = 0%). The subgroup difference approached significance (Q = 3.24, df = 1, *p* = 0.072), suggesting that smaller, less methodologically rigorous trials may overestimate treatment effects.

In the NIV modality subgroup analysis, CPAP-only studies (7 studies, *n* = 527) showed RR 0.68 (95% CI 0.43–1.07; I^2^ = 0%) and BiPAP or mixed-modality studies (7 studies, *n* = 1440) showed RR 0.79 (95% CI 0.58–1.08; I^2^ = 0%).

In the clinical setting, subgroup analysis, emergency department, and prehospital studies (8 studies, *n* = 1699) showed RR 0.80 (95% CI 0.61–1.06; I^2^ = 0%), while ICU/CCU studies (6 studies, *n* = 268) showed RR 0.56 (95% CI 0.32–0.97; I^2^ = 0%). The subgroup difference was not statistically significant (Q = 1.52, df = 1, *p* = 0.22).

### 3.8. Sensitivity Analyses

Sensitivity analyses for hospital mortality were prespecified and supported the robustness of the primary findings. Full results are presented in Supplementary Table S9. In a series of analyses in which we removed the largest trial [13] from the meta-analysis (1069 patients; 54.4% of total participants), the results showed a statistically significant pooled effect (RR 0.62; 95% CI 0.42–0.90; *p* = 0.012; I^2^ = 0%) after excluding 3CPO. When we removed studies with zero events ([20], *n* = 37; [21], *n* = 26), the results were nearly identical to our primary analysis (RR 0.74; 95% CI 0.57–0.96; *p* = 0.024; I^2^ = 0%). Fixed-effect models compared to random-effects models: both fixed- and random-effects models estimated the RR at 0.75 (95% CI 0.58–0.96); this is as expected because there was no heterogeneity (τ^2^ = 0). Sequential leave-one-out analysis: removing each study did not change the results of our meta-analysis. The estimates ranged from 0.62 [13] to 0.78 [22]; all estimates remained below 1.0, and none of the individual studies exerted disproportionate influence on the overall conclusion. Full results of all sensitivity and subgroup analyses are presented in Supplementary Table S9.

### 3.9. Publication Bias

Visual inspection of the funnel plot (Figure S3) revealed asymmetry, with small studies showing larger treatment effects than expected based on the overall estimate. Egger’s regression test [26] confirmed significant asymmetry (intercept = −1.17, SE = 0.38, *p* = 0.007), suggesting small-study effects.

The Duval and Tweedie trim-and-fill analysis [27] imputed three potentially missing studies on the right side of the funnel plot (favoring control). The adjusted pooled estimate accounting for publication bias was RR 0.82 (95% CI 0.64–1.05), which crossed the null and was no longer statistically significant. The presence of funnel plot asymmetry suggests that the true effect of NIV on mortality may be smaller than the point estimate of RR 0.75, potentially approaching the null in the broader ACPE patient population. However, the trim-and-fill method assumes that asymmetry is entirely due to publication bias rather than other sources of small-study effects (e.g., clinical heterogeneity, lower methodological quality in smaller trials) [44].

### 3.10. Certainty of Evidence

The GRADE Summary of Findings is presented in Table 2. For hospital mortality, evidence certainty was rated as moderate [28]. The evidence was not downgraded for risk of bias (67.5% of participants from low-risk studies), inconsistency (I^2^ = 0%), or indirectness (direct comparison in the target population). Although the total sample size (*n* = 1967) did not meet the calculated optimal information size (*n* ≈ 2700), evidence was not downgraded for imprecision because the 95% CI excluded the null (RR = 1.0) and the 95% prediction interval (0.56–0.98) also favored NIV; however, the OIS shortfall represents an additional source of uncertainty. Evidence was downgraded by 1 level for publication bias based on a significant Egger’s test and a trim-and-fill analysis, suggesting potential overestimation of the effect. The detailed GRADE evidence profile is presented in Supplementary Table S2.

For endotracheal intubation, evidence certainty was also rated as moderate. Evidence was downgraded by 1 level for risk of bias because blinding the NIV intervention was not possible, and intubation decisions were subjective and potentially influenced by knowledge of treatment allocation. Evidence was not downgraded for inconsistency (I^2^ = 21%), indirectness, imprecision, or publication bias. In summary, there is moderate certainty evidence that NIV reduces hospital mortality (RR 0.75, 95% CI 0.58–0.96) and endotracheal intubation (RR 0.49, 95% CI 0.35–0.68) compared with standard oxygen therapy in adults with acute cardiogenic pulmonary edema. The mortality benefit appears to be associated with ACS prevalence at the study level, with greater benefit observed in populations with higher ischemic burden; however, individual-patient-level effect modification has not been confirmed.

## 4. Discussion

### 4.1. Principal Findings

The results of our systematic review and meta-analysis of 14 RCTs involving 1967 patients demonstrated that NIV can reduce hospital mortality in ACPE (Relative Risk 0.75, 95% Confidence Interval 0.58–0.96; *p* = 0.022). There was no evidence of statistical heterogeneity (I^2^ = 0%, τ^2^ = 0, Q = 11.57, df = 13, *p* = 0.56). Although our results are consistent with those reported by Berbenetz et al., who conducted a Cochrane review (Relative Risk 0.65, 95% Confidence Interval 0.51–0.82) [9], our primary contribution is to translate a qualitative observation into a quantitative predictive model. In addition to the pooled estimates, we conducted random-effects meta-regressions (using restricted maximum likelihood estimation) to assess whether ACS rates were associated with RR (mortality risk) across studies. Our analysis indicated a significant negative relationship between the RRs and the ACS rate (β_1_ = −0.023 per 1% increase in the ACS proportion, standard error = 0.007, p = 0.008). The amount of explained variance in the RRs using ACS rates was 46.2% (R^2^ = 46.2%). While the pooled analyses demonstrated I^2^ = 0%, the statistically significant β_1_ value indicated that ACS prevalence accounted for systematic variance in the effect sizes, which would otherwise have been ascribed to sampling error. The observed homogeneity probably resulted from a combination of the statistical predominance of 3CPO (with ~54% of total weight) and the low power of Cochran’s Q test when there are <20 studies [18] and the large confidence limits of the remainder of the smaller trials (*n* = 23–130), rather than clinical homogeneity. It was also notable that the meta-regression R^2^ of 46.2% indicated that almost half of the variance among the studies was attributable to ACS prevalence. This variance would remain unobserved within an I^2^ framework.

The equilibrium point—the ACS prevalence at which the predicted log(RR) equals 0—was calculated as—β_0_/β_1_ = 14.1%. Below this threshold, NIV confers no detectable mortality benefit; above it, the benefit increases with increasing ACS prevalence.

This equilibrium point occurs just within the lower limit of ACS rates in the studies included in the meta-analyses (14.6–100%). Any extrapolation beyond this point should be done with appropriate caution. Each 10-percentage-point increase in ACS prevalence corresponds to an approximately 21% reduction in mortality risk (e^−0.23^ = 0.79), demonstrating a clinically meaningful dose–response relationship.

### 4.2. From Qualitative Observation to Quantitative Framework

Weng et al. first identified ischemic etiology as a potential effect modifier, demonstrating an average 17.4% reduction in risk ratio (95% CI 0.7–31.2%) per 10% increase in ischemic proportion [14]. However, critical questions remained: at what ACS prevalence threshold does the mortality benefit emerge, and can the neutral 3CPO result be quantitatively reconciled with the overall evidence? This foundational observation established ischemic etiology as a potential effect modifier but left critical questions unanswered: At what point does the ACS prevalence threshold determine whether the mortality benefit of NIV emerges? How much additional benefit accrues with each incremental increase in ACS prevalence? And can the neutral result of 3CPO be quantitatively reconciled with the overall evidence base? Our analysis extends the previous framework in three methodologically important ways. First, we operationalized the ACS Index using strict temporal criteria from the 2018 Fourth Universal Definition of Myocardial Infarction [43], which require acute ischemic events to be confirmed within 0–72 h of admission. This distinction is critical: Weng et al. [14] categorized studies by “ischemic etiology,” which conflated acute coronary syndromes (acute MI, unstable angina, new-onset ischemia) with chronic ischemic heart disease and prior myocardial infarction as precipitants of decompensation. In contrast, our ACS Index captures only acute ischemic events occurring within the 0–72 h window from presentation—a temporal criterion derived from the Fourth Universal Definition [43] framework. Diagnoses were accepted as reported by each study’s original investigators, using their contemporaneous biomarker criteria.

For Gray et al. [13], the enzyme-based (CK-MB) diagnostic threshold applied in the WHO 1971 criteria [45] (27%) was selected over the troponin-based ESC/ACC 2000 threshold (51.5%) to maintain consistency with the biomarker methodology used in the majority of included trials (Supplementary Table S7). This methodological refinement addresses a fundamental confound: a patient with prior MI three years ago whose ACPE is precipitated by hypertensive crisis represents a pathophysiologically distinct entity from a patient experiencing acute STEMI with secondary pulmonary edema. The Weng [14] categorization grouped both as “ischemic etiology,” diluting the signal from acute ischemia. Our strict temporal criterion isolates the acute ischemic phenotype, in which NIV’s hemodynamic benefits most directly address the underlying pathophysiology. This inclusive approach to defining the ACS Index—encompassing AMI, unstable angina, and acute myocardial ischemia—is consistent with the proposed “Myocardial Ischemic Syndromes” classification by Boden et al. [46], which defines ACS (STEMI, NSTEMI, and UA) as an important subgroup of acute myocardial ischemic syndromes (AMIS). This supports the concept that all acute ischemic events—not just myocardial infarction—constitute the clinically relevant construct for evaluating treatment effects.

Second, weighted meta-regression with inverse-variance weighting enables formal hypothesis testing rather than visual subgroup comparison. Third, the equilibrium point concept provides a quantitative framework for a previously qualitative observation. The convergence of our findings with the Weng observation across independent analytical approaches strengthens confidence in the underlying biological relationship. Consistent findings were also reported by Mariani et al. [47], whose meta-analysis of 34 RCTs (*n* = 3041) demonstrated that both CPAP (RR 0.63, 95% CI 0.44–0.89) and BiPAP (RR 0.73, 95% CI 0.55–0.97) significantly reduced mortality compared with standard therapy, providing additional triangulation.

Moreover, the Cochrane review identified a potentially convergent finding: patients presenting with eucapnia (PaCO_2_ ≤ 45 mmHg) demonstrated significantly greater mortality benefit from NIV (RR 0.41, 95% CI 0.27–0.63; *n* = 581, 10 studies) compared to hypercapnic patients (RR 0.82, 95% CI 0.61–1.10; test for subgroup differences *p* = 0.005) Notably, the eucapnia RR of 0.41 closely approximates what our meta-regression would predict for a high-ACS population, while the hypercapnia RR of 0.82 aligns with predictions for low-ACS populations near the equilibrium threshold [9]. This eucapnia phenotype may correlate with acute, sudden-onset pulmonary edema characteristic of ACS-precipitated respiratory failure. Pathophysiologically, acute MI causes a rapid decline in left ventricular ejection fraction, leading to flash pulmonary edema, triggering compensatory hyperventilation with resultant eucapnia or even hypocapnia. In contrast, chronic heart failure with gradual decompensation allows respiratory adaptation and the development of hypercapnia. This mechanistic link and numerical concordance provide a hypothesis-generating observation supporting potential correlation between the eucapnia phenotype and ACS prevalence. However, direct validation in studies reporting both variables simultaneously is needed.

### 4.3. ACS Index Operationalization: The Exclusion Criteria Paradox

A methodological challenge in constructing the ACS Index arose from the paradox of exclusion criteria. Many trials explicitly excluded patients with “acute myocardial infarction” at enrollment [20,41]; yet, they subsequently reported proportions of patients with MI confirmed during hospitalization. This apparent contradiction reflects the temporal evolution of diagnosis: at randomization, patients presented with acute pulmonary edema and were excluded if STEMI was suspected on initial ECG; however, biomarker confirmation (CK-MB elevation in most trials, as troponins were not yet widely available) occurred 6–24 h post-admission.

We addressed this paradox by using the 0–72 h temporal window from the Fourth Universal Definition as a conceptual guide to determine whether reported diagnoses reflected acute ischemic events contemporaneous with the index ACPE episode. When a patient was enrolled without suspected MI but subsequently confirmed with MI within 0–72 h, the acute ischemia likely precipitated the pulmonary edema rather than representing an independent coincident event. The exclusion criterion in these trials excluded suspected STEMI requiring emergent reperfusion, not subsequent MI of any type. This interpretation is consistent with the clinical reality that NSTEMI and Type 2 MI frequently present with acute pulmonary edema as the dominant symptom, with ischemia recognized only after biomarker confirmation. The temporal window of 0–72 h is based on the Fourth Universal Definition of Acute Myocardial Infarction and aligns with the typical hospitalization period for ACPE in the included trials. Events beyond this window are more likely to represent hospital-acquired complications rather than the precipitating cause.

### 4.4. Methodological Rationale for Stricter Inclusion Criteria

Our analysis includes 14 trials compared to 24 in the Cochrane review by Berbenetz et al. [9] and 31 in the meta-analysis by Weng et al. [14]. This reduction reflects a deliberate methodological choice: meta-regression examining effect modification requires reliable covariate data from every included study. Trials lacking extractable ACS prevalence [33] were excluded, not because they failed quality assessment but because they could not contribute to the effect modification analysis that constitutes our primary research question. Similarly, [30] compared the timing of CPAP initiation (immediate versus 15-min delay) in both groups receiving CPAP—a design that cannot assess NIV versus standard care—and was appropriately excluded. This represents a fundamental trade-off between comprehensiveness and analytical precision. The Cochrane reviews appropriately prioritized comprehensiveness to establish pooled efficacy across diverse populations. Our work prioritizes precision in determining whether ACS prevalence modifies treatment effects and in quantifying that modification. Notably, no significant differences exist between CPAP and BiPAP for either mortality or intubation (test for subgroup differences *p* = 0.64 and *p* = 0.30, respectively) [9], supporting the validity of pooling NIV modalities in our data. A detailed comparison of our methodology and findings with prior meta-analyses is presented in Supplementary Table S10.

### 4.5. The STEMI Evidence Gap: Systematic Exclusion of Highest-Risk Patients

Among the included trials, [20] excluded patients with “AMI requiring thrombolysis,” effectively removing STEMI patients eligible for fibrinolytic therapy [41]. Conducted in the prehospital setting, the study excluded patients with “suspected STEMI.”

Most consequentially, the 3CPO trial—which randomized 1069 patients across 26 UK emergency departments—specifically excluded patients with “myocardial infarction with ST-elevation for which primary PCI was planned.” In contemporary practice, this criterion excludes STEMI patients who qualify for primary percutaneous coronary intervention. The establishment of primary PCI as the standard of care following DANAMI-2 and PRAGUE-2 [48,49] in the early 2000s created a paradox: patients most likely to benefit from NIV, based on our meta-regression—those with acute transmural ischemia—are systematically excluded from pragmatic ED-based trials because they require emergent activation of the catheterization laboratory.

This well-defined patient population has, to date, never undergone an NIV investigation using randomized controlled trials. However, observational data suggests significant benefits: The study by Qu et al. [15] revealed a 34% reduction in mortality (HR = 0.674) in a pure ACS cohort; whereas the only RCT that enrolled solely AMI patients [39], yielded the largest estimate in our meta-analysis (RR = 0.14; 95% CI = 0.02–0.98), and both of these studies support the necessity for prospective clinical trials in STEMI patients with acute pulmonary edema.

### 4.6. Addressing Potential Confounding by Study Size

A legitimate methodological concern is whether our finding reflects effect modification or confounding due to study size. Smaller trials tended to have higher ACS prevalence and showed stronger NIV effects, while the largest trial [13] had moderate ACS prevalence (27%) and showed no mortality benefit. Egger’s test confirmed significant funnel plot asymmetry (intercept = −1.17, *p* = 0.007), indicating potential small-study effects [26].

Importantly, Weng et al. explicitly noted this same phenomenon in 3CPO: “Only about 20% of trial patients had myocardial ischemia or infarction as the cause of their pulmonary edema—a baseline characteristic associated with greater estimates of effect in this meta-analysis” [14]. The discrepancy between the reported ~20% and our extracted 27% ACS prevalence reflects definitional differences: we used the WHO 1971 definition of acute myocardial infarction within the 0–72 h temporal framework, which captures a broader spectrum of acute ischemic events than the narrower “ischemic etiology as precipitant” categorization used by Weng [14]. This standardized approach aligns with contemporary diagnostic criteria and enables consistent cross-study comparison. Meta-analysis findings are reflected in this finding from our meta-regression: at an ACS prevalence of 27%, the model predicts RR = 0.75 (95% CI 0.58–0.97) for an observed 3CPO mortality RR of 0.97 (95% CI 0.66–1.43; NIV 67/702 [9.5%] vs. O2 36/367 [9.8%]). The models’ point estimates differ; however, their confidence intervals overlap substantially, and three factors explain the difference. The first factor is that 3CPO was designed to detect a 6% absolute difference in mortality (9% vs. 15%). Therefore, the 25% relative risk reduction at the observed baseline of 9.8% represents a 2.5% absolute difference, which is below the study’s detection limit. The second factor is that 15% of patients who received standard oxygen therapy received rescue NIV within 2 h after randomization, and the intubation rates were identical (3% vs. 3%), compared with the 27% vs. 12% rates reported in prior meta-analyses. Therefore, the 3CPO investigators stated that they effectively studied first-line delayed NIV, not NIV versus no NIV [50]. The third factor is our pooled estimate across all 14 studies, which also yields an RR of 0.75 (95% CI 0.58–0.96), the same RR that the meta-regression independently predicts at the given ACS prevalence. While small-study effect inflation of the pooled estimate of ACS prevalence and the meta-regression slope may be considered, the issue is whether the covariation (between ACS prevalence and treatment size) of the characteristics of smaller trials (in reporting both ACS prevalence and treatment size) will improve the apparent dose–response relationship.

Several considerations argue against dismissing our findings as a mere artifact. First, weighted meta-regression using inverse-variance weights inherently accounts for study precision—larger studies exert proportionally greater influence on the regression slope. The persistence of a significant association (*p* = 0.008) after such weighting suggests the relationship is not driven solely by small, imprecise studies. Second, the biological plausibility of differential NIV benefit in acute ischemia provides mechanistic grounding independent of statistical patterns. Third, the consistency between our quantitative meta-regression and the qualitative observation—derived from different analytical approaches—constitutes triangulation evidence.

Notably, Weng et al. [14] themselves documented significant publication bias (Begg test, *p* = 0.002), yet their observation of differential benefit by ischemic etiology remains widely accepted. Methodological consistency requires that our quantitative extension be evaluated on the same substantive grounds.

We recognize that low-risk-of-bias trials alone (4 studies, *N* = 1327) were insufficient to achieve statistical significance for mortality reduction (Relative Risk [RR] 0.88; 95% Confidence Interval [CI]: 0.64–1.22), whereas high-risk-of-bias trials showed a larger effect size (RR 0.54). While the low-risk-of-bias subgroup was primarily composed of 3CPO (N = 1069, accounting for approximately 80% of this subgroup), the between-subgroup test for effect modification did not reach statistical significance (Q = 3.24, df = 1, *p* = 0.072). Therefore, it appears that the pattern we observed above may be influenced by the prevalence gradient of ACS in smaller, earlier trials- rather than solely by inflated effect sizes due to bias. Nevertheless, we cannot definitively exclude confounding between small-study effects and effect modification. An individual-patient data meta-analysis would enable within-study examination of the ACS-outcome relationship, eliminating the ecological bias inherent in study-level meta-regression. We have accordingly downgraded our GRADE certainty assessment for the mortality outcome due to concerns about publication bias.

### 4.7. Biological Plausibility: Hemodynamic Mechanisms

The hemodynamic effects of positive-pressure ventilation provide a plausible mechanistic rationale for greater NIV benefit in ACS-precipitated ACPE. During acute myocardial ischemia, the oxygen supply-demand balance becomes critical. Coronary blood flow occurs predominantly during diastole, and the ischemic myocardium has limited reserve for increasing oxygen extraction (baseline extraction ratio 70–80%) [51]. NIV reduces myocardial oxygen demand through synergistic mechanisms: increased intrathoracic pressure reduces left ventricular transmural pressure and afterload; positive end-expiratory pressure optimizes preload; and respiratory muscle unloading eliminates the substantial metabolic cost of labored breathing, which can consume 25–30% of cardiac output during severe respiratory distress [52].

Equally important is what NIV allows patients to avoid. Endotracheal intubation carries significant hemodynamic risks: post-intubation hypotension occurs in approximately 20% of critically ill ICU patients, and cardiac arrest occurs in 0.7–11% of emergency intubations, depending on the level of difficulty [53,54]. For patients with acute coronary syndrome, any hypotensive episode risks extending myocardial injury through reduced coronary perfusion pressure. The consistent reduction in intubation rates demonstrated across meta-analyses (RR 0.49, 95% CI 0.38–0.62; per Berbenetz [9]) may therefore translate into greater mortality benefit in populations where intubation-associated hypotension poses the greatest risk.

### 4.8. Clinical Implications

Our findings suggest that current clinical practice may underestimate the benefit of NIV in high-risk ACS populations and overestimate it in low-risk populations. The 14.1% ACS prevalence equilibrium point provides a clinically informative threshold: in settings where ACS accounts for fewer than 15% of ACPE presentations, the expected mortality benefit from NIV approaches zero. Conversely, in cardiac intensive care units, chest pain centers, and catheterization laboratory settings where ACS predominates, our analysis suggests NIV may confer benefits beyond respiratory support in this population. Contemporary observational data from Qu et al., examining 1,257 ACS patients with acute systolic heart failure (100% ACS prevalence), demonstrated that NIV independently reduced one-year all-cause mortality (HR 0.674, 95% CI 0.458–0.991, *p* = 0.045) and significantly decreased in-hospital intubation rates (3.50% vs. 6.36%, *p* = 0.027) [15]. These findings in a pure ACS population are directionally consistent with our hypothesis, though the observed HR of 0.674 is more conservative than the value predicted by the strict extrapolation of our regression for 100% ACS prevalence. This discrepancy likely reflects the inherent uncertainty of extrapolating beyond the observed data range (ACS prevalence in included RCTs ranged from 14.6% to 100%, with only one study at 100%), differences between hazard ratios and risk ratios, and unmeasured confounding in the observational design. Furthermore, the Qu study employed a historical control design (control group 2010–2014; NIV group 2020–2022), and secular trends in standard care over the ~6-year gap between the study periods may confound the observed association.

### 4.9. Limitations

Several limitations affect interpretation. First, meta-regression operates at the study level rather than the patient level, creating vulnerability to ecological fallacy—the relationship observed between ACS prevalence and NIV benefit across studies may not hold for individual patients within studies. Second, our ACS Index relies on reported data that varied in completeness across studies; biomarker confirmation relied on CK-MB in most trials rather than contemporary high-sensitivity troponins, potentially underestimating the true ACS prevalence. Third, the R^2^ of 46.2% indicates that ACS prevalence explains less than half of the systematic variation in treatment effects; other factors including NIV pressure settings (CPAP 2.5–20 cm H_2_O; BiPAP IPAP 8–20, EPAP 3–10 cm H_2_O across trials), time from symptom onset to NIV initiation, and background pharmacotherapy (nitrates, diuretics) may contribute to residual variation. Furthermore, ACS prevalence may be associated with other trial-level characteristics—such as illness severity, clinical environment, era of treatment, and availability of treatments for reperfusion—that can independently affect outcomes and therefore complicate causal inference.

Fourth, the systematic exclusion of STEMI patients from major trials means conclusions about this population necessarily extrapolate from studies with lower ACS prevalence. Fifth, the included trials span 1985–2011, during which both NIV technology and ACPE management evolved substantially. Sixth, the 0.5 continuity correction applied to studies with zero events (per Cochrane guidelines) may modestly affect effect estimates in small studies, though sensitivity analysis excluding these studies did not materially alter our conclusions. Seventh, allocation concealment was unclear or inadequate in several trials, and subgroup analyses by Weng et al. [14] suggested larger effect estimates in studies with adequate concealment.

This review was conducted in accordance with the PROSPERO protocol (CRD420251142245). The ACS Index operationalization and equilibrium-point derivation constitute analytical refinements of the prespecified hypothesis, and all amendments were recorded before data extraction.

### 4.10. Future Research Directions

The evidence gaps we have identified in this systematic review indicate that many areas warrant further research. These findings support the need for a randomized clinical trial of NIV in patients with STEMI complicated by acute pulmonary edema undergoing primary PCI, powered to evaluate 30-day mortality and stratified by Killip classification. Such a trial would directly address the paradox that, according to our meta-regression model, patients who appear to derive the greatest benefit from NIV have been systematically excluded from previous studies. Individual participant data meta-analyses, including all existing RCTs, may offer an opportunity to study how NIV has different effects at the individual level than previously thought and eliminate ecological bias. Registry-based analyses linking NIV to outcomes in contemporary STEMI populations could yield hypotheses for future studies. Notably, Giordana et al. recently demonstrated that CPAP during PCI in AMI patients with cardiogenic shock was feasible, well-tolerated, and may even facilitate the procedure [55]. These preliminary findings suggest that NIV can be safely implemented during acute coronary revascularization.

### 4.11. Conclusions

This meta-analysis synthesizes diverse data into a predictive model: NIV in acute cardiogenic pulmonary edema shows a dose–response relationship with the prevalence of ACS, shifting from a null effect on mortality to a statistically significant reduction at the same equilibrium point (14.1%). By applying the 0–72 h temporal framework from the Fourth Universal Definition of Myocardial Infarction [43] to distinguish acute ischemia from chronic coronary disease, we were able to explain why landmark trials conducted in low-ACS populations showed no mortality benefit, whereas those enrolling high-ACS populations demonstrated substantial benefits. NIV may provide benefits that extend beyond ventilatory support in respiratory failure precipitated by ACS. The next crucial step will be randomized controlled trials in the population most likely to benefit, as identified by our study (STEMI patients with acute pulmonary edema). Paradoxically, this group is currently excluded from the evidence base.

## Figures and Tables

**Table 1:** Characteristics of included randomized controlled trials examining non-invasive ventilation versus standard oxygen therapy in acute cardiogenic pulmonary edema (*n* = 14 studies, *N* = 1967 participants).

Study	Country	Setting	*N*	NIV/Control	Age (y)	Male (%)	pH	ACS (%)
Räsänen et al., 1985 [36]	Finland	ICU	40	20/20	74	32	7.35	47.5
Bersten et al., 1991 [19]	Australia	ICU	39	19/20	76	33	7.17	43.6
Lin et al., 1995 [37]	Taiwan	CCU	100	50/50	72	90	NR	21.0
Takeda et al., 1997 [38]	Japan	ICU	30	15/15	66	73	NR	36.7
Takeda et al., 1998 [39]	Japan	CCU	22	11/11	74	77	NR	100.0
Masip et al., 2000 [20]	Spain	ICU	37	19/18	77	52	7.30	32.4
Levitt 2001 [42]	USA	ED	38	21/17	68	33	NR	23.7
Park et al., 2001 [21]	Brazil	ED	26	16/10	69	41	7.33	34.6
Nava et al., 2003 [34]	Italy	ED	130	65/65	78	48	7.25	16.9
Crane et al., 2004 [40]	UK	ED	60	40/20	75	40	7.20	30.0
L’Her et al., 2004 [35]	France	ED	89	43/46	84	42	7.31	14.6
Park et al., 2004 [22]	Brazil	ED/ICU	80	54/26	64	45	7.33	43.8
Gray et al., 2008 [13]	UK	ED	1069	702/367	78	43	7.21	27.0
Ducros et al., 2011 [41]	France	EMS	207	107/100	80	41	7.30	17.4
Total/Weighted Mean	10 countries	—	1967	1182/785	77	46	7.24	27.1

Abbreviations: ACS, acute coronary syndrome; CCU, coronary care unit; ED, emergency department; EMS, emergency medical services; ICU, intensive care unit; NIV, non-invasive ventilation; NR, not reported. Age and male percentage are the study’s mean values. pH represents baseline arterial pH. ACS prevalence is defined as the proportion of patients with acute myocardial infarction or an acute ischemic etiology within 0–72 h, according to the Fourth Universal Definition of Myocardial Infarction criteria [43].

**Table 2:** GRADE Summary of Findings: Non-invasive Ventilation Compared to Standard Oxygen Therapy for Acute Cardiogenic Pulmonary Edema. Patient or population: Adults with acute cardiogenic pulmonary edema|Setting: Emergency department, ICU, CCU, or prehospital Intervention: Non-invasive ventilation (CPAP or BiPAP)|Comparison: Standard oxygen therapy.

Outcomes	Participants	Certainty Assessment					Relative Effect	Anticipated Absolute Effects		Certainty
		Risk of Bias	Inconsistency	Indirectness	Imprecision	Publication Bias		Standard O_2_	Difference	
Hospital mortality (227 events)	1967 (14 RCTs)	○	○	○	○	⊖ ᵃ	RR 0.75 (0.58–0.96)	141 per 1000	35 fewer (6 to 59 fewer)	⊕⊕⊕○ MODERATE
Endotracheal intubation (185 events)	1967 (14 RCTs)	⊖ ᵇ	○	○	○	○	RR 0.49 (0.35–0.68)	146 per 1000	76 fewer (47 to 96 fewer)	⊕⊕⊕○ MODERATE

GRADE certainty ratings: ⊕⊕⊕○ Moderate = moderately confident. ○ = not downgraded; ⊖ = downgraded one level. ᵃ Publication bias: Downgraded one level. Egger’s regression test showed significant funnel plot asymmetry (intercept = −1.17, SE = 0.38, *p* = 0.007), suggesting potential small-study effects favoring NIV. ᵇ risk of bias: Downgraded one level. The inability to blind the NIV intervention may influence clinical decisions regarding intubation, as this outcome is subjective and determined by treating physicians. Abbreviations: BiPAP, bilevel positive airway pressure; CCU, coronary care unit; CI, confidence interval; CPAP, continuous positive airway pressure; GRADE, Grading of Recommendations Assessment, Development and Evaluation; ICU, intensive care unit; NIV, non-invasive ventilation; RCT, randomized controlled trial; RR, risk ratio.

## Data Availability

All data generated or analyzed during this study are included in this published article and its Supplementary Information Files.

## Supplementary Materials

The following supporting information can be downloaded at: https://www.mdpi.com/article/10.3390/jcdd13030135/s1, Table S1: Leave-one-out sensitivity analysis for the mortality outcome; Table S2: GRADE evidence profile; Table S3: Subgroup analysis for hospital mortality; Table S4: Characteristics of excluded studies (Refs: [56,57,58,59,60,61,62,63,64,65,66,67,68,69,70,71,72,73,74,75,76,77,78,79,80]); Table S5: Search strategy for all databases; Table S6: PRISMA 2020 checklist; Table S7: ACS Index extraction methodology; Table S8: Meta-regression analysis; Table S9: Sensitivity and subgroup analyses for hospital mortality; Table S10: Comparison with prior meta-analyses; Figure S1: Leave-one-out sensitivity analysis forest plot; Figure S2: Risk of bias summary plot; Figure S3: Funnel plot; Figure S4: Risk of bias traffic-light plot.

## Abbreviations

The following abbreviations are used in this manuscript:

3CPO	3 Interventions in Cardiogenic Pulmonary Oedema trial
ACPE	acute cardiogenic pulmonary edema
ACS	acute coronary syndrome
AMI	acute myocardial infarction
BiPAP	bilevel positive airway pressure
CCU	coronary care unit
CI	confidence interval
CPAP	continuous positive airway pressure
df	degrees of freedom
DL	DerSimonian–Laird
ED	emergency department
EMS	emergency medical services
GRADE	Grading of Recommendations Assessment, Development and Evaluation
I^2^	heterogeneity statistic
ICU	intensive care unit
MeSH	Medical Subject Headings
MI	myocardial infarction
NIV	non-invasive ventilation
NNT	number needed to treat
NSTEMI	non-ST-elevation myocardial infarction
OIS	optimal information size
PaCO_2_	partial pressure of arterial carbon dioxide
PCI	percutaneous coronary intervention
PRISMA	Preferred Reporting Items for Systematic Reviews and Meta-Analyses
PROSPERO	International Prospective Register of Systematic Reviews
Q	Cochran’s statistic
RCT	randomized controlled trial
REML	restricted maximum likelihood
RoB	risk of bias
RR	risk ratio
RRR	relative risk reduction
SE	standard error
STEMI	ST-elevation myocardial infarction
UA	unstable angina
τ^2^	between-study variance
