# Supplementary material for: Effect Modification by Acute Coronary Syndrome Prevalence on Non-Invasive Ventilation Efficacy in Acute Cardiogenic Pulmonary Edema: A Systematic Review and Meta-Analysis of Randomized Controlled Trials
Source: J Cardiovasc Dev Dis. 2026 Mar 12;13(3):135. doi: 10.3390/jcdd13030135 (PMC13026454; doi:10.3390/jcdd13030135)
Supplement: Supplementary file 1 [file jcdd-13-00135-s001.zip › jcdd-4148745-supplementary.pdf]

Supplementary Materials

Effect Modification by Acute Coronary Syndrome Prevalence on Noninvasive Ventilation Efficacy in Acute Cardiogenic Pulmonary Edema: A Systematic Review and Meta-Analysis of Randomized Controlled Trials

Table S1. Leave-one-out sensitivity analysis for the mortality outcome.

Study Omitted	n	Pooled RR	95% CI	I^2 (%)	p-value
Räsänen 1985 [36]	40	0.76	0.59–0.98	0.0	0.035
Bersten 1991 [19]	39	0.75	0.58–0.97	0.0	0.029
Lin 1995 [37]	100	0.75	0.58–0.97	0.0	0.028
Takeda 1997 [38]	30	0.75	0.59–0.97	0.0	0.029
Takeda 1998 [39]	22	0.77	0.60–0.99	0.0	0.041
Masip 2000 [20]	37	0.75	0.58–0.97	0.0	0.028
Levitt 2001 [42]	38	0.74	0.58–0.96	0.0	0.023
Park 2001 [21]	26	0.74	0.58–0.95	0.0	0.020
Nava 2003 [34]	130	0.75	0.58–0.98	0.0	0.032
Crane 2004 [40]	60	0.77	0.60–1.00	0.0	0.050
L'Her 2004 [35]	89	0.72	0.55–0.94	0.0	0.018
Park 2004 [22]	80	0.78	0.60–1.01	0.0	0.056
Gray 2008 [13]	1069	0.61	0.44–0.85	0.0	0.004
Ducros 2011 [41]	207	0.74	0.57–0.96	0.0	0.024
**Overall (k = 14)**	**1967**	**0.75**	**0.58–0.96**	**0.0**	**0.022**

Abbreviations: *CI, confidence interval; I^2, heterogeneity statistic; RR, risk ratio.*

Note: Each row displays the pooled effect estimate after excluding the corresponding study. The overall estimate (k = 14) represents the primary meta-analysis, including all studies. Risk ratios < 1.0 favor noninvasive ventilation. Note on borderline result: When Crane 2004 [40] is excluded, p = 0.050 (exact model output, 3 decimal places) and the 95% CI upper bound simultaneously reaches 1.00 (0.60–1.00), consistent with a borderline result; this leave-one-out estimate should be interpreted cautiously.

Table S2. GRADE Evidence Profile: Noninvasive Ventilation Compared to Standard Oxygen Therapy for Acute Cardiogenic Pulmonary Edema

Patient or population: Adults with acute cardiogenic pulmonary edema

Setting: Emergency department, intensive care unit, coronary care unit, or prehospital

Intervention: Noninvasive ventilation (CPAP or BiPAP)

Comparison: Standard oxygen therapy (face mask or nasal cannula)

Baseline risk source: Control group of included studies

Outcomes	No. of participants (studies)	Risk of bias	Inconsistency	Indirectness	Imprecision	Other considerations	Relative effect (95% CI)	Risk with standard O_2	Risk difference with NIV (95% CI)	Certainty	Importance
Hospital mortality (in-hospital) 227 events	1967 (14 RCTs)	Not serious[a]	Not serious (I^2 = 0%)	Not serious	Not serious[b]	Publication bias (serious)[c]	**RR 0.75 (0.58 to 0.96)**	141 per 1000	35 fewer per 1000 (6 to 59 fewer)	⊕⊕⊕◯ **MODERATE**	CRITICAL
Endotracheal intubation (in-hospital) 185 events	1967 (14 RCTs)	Serious[d]	Not serious (I^2 = 21%)	Not serious	Not serious[b]	Not formally assessed[e]	**RR 0.49 (0.35 to 0.68)**	146 per 1000	76 fewer per 1000 (47 to 96 fewer)	⊕⊕⊕◯ **MODERATE**	CRITICAL

Explanations

[a] **Risk of bias (mortality):** Not downgraded. Although blinding was not possible, mortality is an objective (hard) outcome unlikely to be influenced by knowledge of treatment allocation.

[b] **Imprecision:** Not downgraded. Optimal information size (OIS) was met with 1967 participants and adequate events. The 95% CI excludes a no-effect value (RR = 1.0) for both outcomes.

[c] **Publication bias (mortality):** Downgraded one level. Egger's test: intercept = −1.17, SE = 0.38, p = 0.007. Funnel plot asymmetry suggests small-study effects.

[d] **Risk of bias (intubation):** Downgraded one level. Blinding not possible. Intubation is a subjective outcome determined by treating physicians; lack of blinding may influence clinical decision-making.

[e] **Publication bias (intubation):** Not formally assessed for secondary outcome. No upgrade or downgrade applied.

[f] ⊕⊕⊕○ Moderate = moderately confident.

Upgrade factors considered

Large effect: Not applied. RR for intubation (0.49) approaches but does not meet the threshold (RR < 0.5). Dose-response: Not applicable. Plausible confounding: Not applied.

Outcome definitions

Hospital mortality: Death from any cause during index hospitalization.

Endotracheal intubation: Need for invasive mechanical ventilation during index hospitalization.

Additional information: NNT mortality: 29 (17 to 167). NNT intubation: 13 (10 to 21). Heterogeneity mortality: I^2 = 0%, τ^2 = 0, Q = 11.57 (df = 13, p = 0.56). Heterogeneity intubation: I^2 = 21%, τ^2 = 0.08, Q = 16.5 (df = 13, p = 0.22).

Abbreviations: *BiPAP, bilevel positive airway pressure; CI, confidence interval; CPAP, continuous positive airway pressure; df, degrees of freedom; GRADE, Grading of Recommendations Assessment, Development and Evaluation; I^2, heterogeneity; NIV, noninvasive ventilation; NNT, number needed to treat; OIS, optimal information size; Q, Cochran's Q; RCT, randomized controlled trial; RR, risk ratio; SE, standard error; τ^2, between-study variance.*

Table S3. Subgroup Analysis for Hospital Mortality: NIV versus Standard Oxygen Therapy

Subgroup	Studies	N	RR	95% CI	I^2	Subgroup Difference
ACS Prevalence						
ACS >25%	9	1403	**0.65**	**0.47–0.91**	0%	Q=2.89, p=0.089
ACS ≤25%	5	564	0.93	0.61–1.43	0%	
Risk of Bias						
**Low risk**	**4**	**1327**	**0.88**	**0.64–1.22**	**0%**	**Q=3.24, p=0.072**
**High risk**	**10**	**640**	**0.54**	**0.35–0.83**	**0%**	
NIV Modality						
CPAP only	7	527	0.68	0.43–1.07	0%	Q=0.31, p=0.58
BiPAP/mixed	7	1440	0.79	0.58–1.08	0%	
Clinical Setting						
ED/Prehospital	8	1699	0.80	0.61–1.06	0%	Q=1.52, p=0.22
ICU/CCU	6	268	**0.56**	**0.32–0.97**	0%	
**Overall**	**14**	**1967**	**0.75**	**0.58–0.96**	**0%**	**—**

Bold values indicate statistically significant results ($p < 0.05$ or 95% CI excludes 1.0).

Abbreviations: ACS, acute coronary syndrome; BiPAP, bilevel positive airway pressure; CCU, coronary care unit; CI, confidence interval; CPAP, continuous positive airway pressure; ED, emergency department; ICU, intensive care unit; N, number of participants; NIV, noninvasive ventilation; Q, Cochran's Q statistic; RR, risk ratio.

Note: Subgroup differences were tested using the chi-squared test. A p-value < 0.05 indicates significant effect modification. All analyses used random-effects models (DerSimonian-Laird).

Table S4. Characteristics of Excluded Studies

Summary of Exclusions

Exclusion Category	n	Studies
No O_2 control arm (CPAP vs BiPAP only)	12	Mehta, Martin-Bermudez, Cross, Liesching 2003, Bellone 2004, Bellone 2005, Ferrari 2006, Ferrari 2007, Moritz 2007, Ferrari 2009, Fontanella 2010, Liesching 2014
Wrong intervention	3	Plaisance 2007, Bollaert 2002, Sharon 2000
Wrong population	3	Delclaux 2000, Ferrer 2003, Thys 2002
ACS excluded/no data	3	Frontin 2011, El-Refay 2016, Weitz 2007
Incomplete/no AMI%	2	Moritz 2003, Austin 2018
Overlapping cohort	1	Lin 1991
No AMI by arm	1	Kelly 2002
**Total excluded**	**25**	
Reports not retrieved	4	Hao 2002 [77], Li 2005 [78], Zokaei 2016 [79], Agmy/Ghanem 2008 [80]
Reports sought for retrieval	43	
Reports assessed for eligibility	39	= 43 − 4
**Studies included in meta-analysis**	**14**	N = 1967 participants

Reports Not Retrieved (n = 4)

#	Study	N	Reason Not Retrieved
1	Hao 2002 [77]	51	Chinese language publication; full text unavailable
2	Li 2005 [78]	42	Chinese language publication; full text unavailable
3	Zokaei 2016 [79]	120	Not indexed in PubMed; no author response to data request
4	Agmy/Ghanem 2008 [80]	129	Abstract only; data discrepancies with ClinicalTrials.gov registration

Excluded Studies with Reasons (n = 25)

#	Study	N	Category	Reason for Exclusion
1	Mehta 1997 [56]	27	No O_2 control arm	Compared CPAP vs BiPAP without standard oxygen therapy control arm
2	Martin-Bermudez 2002 [57]	80		Compared CPAP vs BiPAP without standard oxygen therapy control; abstract only
3	Cross 2003 [58]	71		Compared CPAP vs BiPAP without standard oxygen therapy control arm
4	Liesching 2003 [59]	27		Compared CPAP vs BiPAP without standard oxygen therapy control; abstract only
5	Bellone 2004 [60]	46		Compared CPAP vs BiPAP without standard oxygen therapy control; ACS excluded
6	Bellone 2005 [61]	36		Compared CPAP vs BiPAP without standard oxygen therapy control; hypercapnic subgroup
7	Ferrari 2006 [76]	106		Compared CPAP vs BiPAP without standard oxygen therapy control arm
8	Ferrari 2007 [62]	52		Compared CPAP vs BiPAP without standard oxygen therapy control arm
9	Moritz 2007 [31]	109		Compared CPAP vs BiPAP without standard oxygen therapy control arm
10	Ferrari 2009 [63]	80		Compared CPAP vs BiPAP without standard oxygen therapy control arm
11	Fontanella 2010 [64]	121		Compared CPAP vs BiPAP without standard oxygen therapy control; abstract only
12	Liesching 2014 [65]	49		Compared CPAP vs BiPAP without standard oxygen therapy control arm
13	Plaisance 2007 [30]	124	Wrong intervention	Timing comparison (early vs delayed CPAP); both arms received CPAP intervention
14	Bollaert 2002 [66]	36		Compared CPAP vs proportional assist ventilation (PAV); abstract only
15	Sharon 2000 [67]	40		Compared BiPAP vs high-dose isosorbide dinitrate IV; different control intervention
16	Delclaux 2000 [68]	42	Wrong population	Mixed acute respiratory failure population; only 4% had ACPE
17	Ferrer 2003 [69]	30		Mixed hypoxemic respiratory failure population; only 17% had ACPE
18	Thys 2002 [70]	20		Mixed population (60% COPD exacerbation); only 8 patients had ACPE
19	Frontin 2011 [32]	122	ACS excluded/no data	AMI excluded at baseline; no ACS outcome data reported during/after intervention
20	El-Refay 2016 [71]	66		AMI and unstable angina excluded; no outcome data reported

#	Study	N	Category	Reason for Exclusion
21	Weitz 2007 [72]	23		Excluded angina and ST-elevation; quasi-RCT (alternating allocation); abstract only
22	Moritz 2003 [73]	30	Incomplete/no AMI%	Pilot study with 30-minute protocol only; no clinical endpoints reported
23	Austin 2018 [74]	50		Early termination due to budget constraints; no AMI prevalence data reported
24	Lin 1991 [75]	55	Overlapping cohort	Same institution as Lin 1995; earlier smaller publication with overlapping cohort
25	Kelly 2002 [33]	58	No AMI by arm	AMI reported only as composite with mortality; not available by treatment arm for meta-regression

Abbreviations: *ACPE, acute cardiogenic pulmonary edema; ACS, acute coronary syndrome; AMI, acute myocardial infarction; BiPAP, bilevel positive airway pressure; COPD, chronic obstructive pulmonary disease; CPAP, continuous positive airway pressure; IV, intravenous; N, sample size;* O_2*, oxygen; PAV, proportional assist ventilation; RCT, randomized controlled trial.*

Note: [74] Austin 2018: a data-sharing request submitted to the corresponding author of Austin 2018 yielded no response. The 2010 publication (Austin et al., BMJ 2010) is listed under reference [74] as the closest available source for this research group. This study was excluded from the quantitative synthesis (Table S4) and was not included in the meta-analysis. [76] Ferrari 2006: this is a short communication (N = 106, as reported in Berbenetz 2019 [9]); the year in the study label reflects the publication year of this report.

Table S5. Search Strategy

Protocol registered: PROSPERO CRD420251142245. *Search date: 1 January 1990 to 31 December 2025. No language restrictions.*

**MEDLINE/PubMed**	
#	**Search Terms**
1	"noninvasive ventilation"[MeSH] OR "non-invasive ventilation"[tiab] OR "noninvasive ventilation"[tiab] OR NIV[tiab]
2	"continuous positive airway pressure"[MeSH] OR "continuous positive airway pressure"[tiab] OR CPAP[tiab]
3	"bilevel positive airway pressure"[tiab] OR BiPAP[tiab] OR BPAP[tiab] OR "bi-level"[tiab]
4	"positive pressure respiration"[MeSH] OR "positive pressure ventilation"[tiab]
5	#1 OR #2 OR #3 OR #4
6	"pulmonary edema"[MeSH] OR "pulmonary oedema"[tiab] OR "pulmonary edema"[tiab] OR "lung edema"[tiab]
7	"heart failure"[MeSH] OR "cardiac failure"[tiab] OR "heart failure"[tiab] OR "acute heart failure"[tiab]
8	"cardiogenic"[tiab] AND ("pulmonary edema"[tiab] OR "respiratory failure"[tiab])
9	#6 OR #7 OR #8
10	"randomized controlled trial"[pt] OR "controlled clinical trial"[pt] OR randomized[tiab] OR randomised[tiab] OR placebo[tiab] OR randomly[tiab] OR trial[tiab]
11	#5 AND #9 AND #10; Filters: Humans, 1990/01/01–2025/12/31
Results	1606 records

**Cochrane Library (CENTRAL)**	
#	**Search Terms**
1	[mh "noninvasive ventilation"] OR (non-invasive ventilation OR noninvasive ventilation OR NIV):ti,ab
2	[mh "continuous positive airway pressure"] OR (CPAP OR continuous positive airway pressure):ti,ab
3	(BiPAP OR BPAP OR bilevel positive airway pressure OR bi-level):ti,ab
4	#1 OR #2 OR #3
5	[mh "pulmonary edema"] OR (pulmonary oedema OR pulmonary edema):ti,ab
6	[mh "heart failure"] OR (heart failure OR acute heart failure OR cardiac failure):ti,ab
7	cardiogenic:ti,ab AND (pulmonary edema OR respiratory failure):ti,ab

8	#5 OR #6 OR #7
9	#4 AND #8; Cochrane Library, Trials, 1990–2025
Results	1149 records

**Embase (Ovid)**	
#	**Search Terms**
1	exp noninvasive ventilation/ OR (non-invasive ventilation OR noninvasive ventilation OR NIV).ti,ab.
2	exp continuous positive airway pressure/ OR (CPAP OR continuous positive airway pressure).ti,ab.
3	(BiPAP OR BPAP OR bilevel positive airway pressure OR bi-level).ti,ab.
4	1 OR 2 OR 3
5	exp lung edema/ OR (pulmonary edema OR pulmonary oedema OR lung edema).ti,ab.
6	exp heart failure/ OR (heart failure OR cardiac failure OR acute heart failure).ti,ab.
7	(cardiogenic adj3 (pulmonary edema OR respiratory failure)).ti,ab.
8	5 OR 6 OR 7
9	exp randomized controlled trial/ OR exp controlled clinical trial/ OR (randomized OR randomised OR placebo OR randomly OR trial).ti,ab.
10	4 AND 8 AND 9; limit to human and yr="1990–2025"
Results	1115 records

Summary of Search Results

Source	Records	Notes
MEDLINE/PubMed	1606	
Cochrane Library (CENTRAL)	1149	
Embase	1115	
CINAHL	504	
LILACS	85	*Added post-hoc*
DARE	12	*Added post-hoc*
WHO ICTRP	213	*Trial registry*
ClinicalTrials.gov	124	*Trial registry*
Total records identified	4808	
Duplicates removed	1441	
Records screened (title/abstract)	3367	
Excluded by title/abstract	3324	
Reports sought for retrieval	43	
Reports not retrieved	4	*No full text available*
Reports assessed for eligibility	39	
Reports excluded with reasons	25	*See Table S4*
Studies included in meta-analysis	14	N = 1967 participants

Abbreviations: *CENTRAL, Cochrane Central Register of Controlled Trials; CINAHL, Cumulative Index to Nursing and Allied Health Literature; CPAP, continuous positive airway pressure; DARE, Database of Abstracts of Reviews of Effects; ICTRP, International Clinical Trials Registry Platform; LILACS, Latin American and Caribbean Health Sciences Literature; MeSH, Medical Subject Headings; NIV, noninvasive ventilation; tiab, title/abstract; WHO, World Health Organization.*

Note: *Screening performed independently by two reviewers (M.T., M.D.) using Covidence software. Disagreements resolved by consensus or third-party adjudication (M.B.). Interrater reliability: Cohen's κ = 0.89.*

Table S6. PRISMA 2020 Checklist

Section and Topic	Item #	Checklist Item		Location Where Item Reported
TITLE	1	Title	Identify the report as a systematic review.	Title
ABSTRACT	2	Abstract	See the PRISMA 2020 for Abstracts checklist.	Abstract
INTRODUCTION	3	Rationale	Describe the rationale for the review in the context of existing knowledge.	Section 1
	4	Objectives	Provide an explicit statement of the objective(s) or question(s) the review addresses.	Section 1
METHODS	5	Eligibility criteria	Specify the inclusion and exclusion criteria for the review and how studies were grouped for the syntheses.	Section 2.2
	6	Information sources	Specify all databases, registers, websites, organisations, reference lists and other sources searched or consulted to identify studies.	Section 2.3; Table S5
	7	Search strategy	Present the full search strategies for all databases, registers and websites, including any filters and limits used.	Section 2.4; Table S5
	8	Selection process	Specify the methods used to decide whether a study met the inclusion criteria of the review.	Section 2.5
	9	Data collection process	Specify the methods used to collect data from reports.	Section 2.6
	10a	Data items	List and define all outcomes for which data were sought.	Sections 2.6, 2.8
	10b	Data items	List and define all other variables for which data were sought.	Section 2.6; Table 1
	11	Study risk of bias assessment	Specify the methods used to assess risk of bias in the included studies.	Section 2.7
	12	Effect measures	Specify for each outcome the effect measure(s) used in the synthesis or presentation of results.	Section 2.8
	13a	Synthesis methods	Describe the processes used to decide which studies were eligible for each synthesis.	Section 2.8
	13b	Synthesis methods	Describe any methods required to prepare the data for presentation or synthesis.	Section 2.8
	13c	Synthesis methods	Describe any methods used to tabulate or visually display results of individual studies and syntheses.	Section 2.8; Figures 2–4; Supplementary Figures S1–S4
	13d	Synthesis methods	Describe any methods used to synthesize results and provide a rationale for the choice(s).	Section 2.8
	13e	Synthesis methods	Describe any methods used to explore possible causes of heterogeneity among study results.	Sections 2.9, 2.10

Checklist adapted from: Page MJ, McKenzie JE, Bossuyt PM, et al. The PRISMA 2020 statement: an updated guideline for reporting systematic reviews. BMJ 2021;372:n71. doi: 10.1136/bmj.n71

Table S7. ACS Index Extraction Methodology for Included Randomized Controlled Trials (k = 14, N = 1967).

Study	N	Original Reported Data (AMI/Ischemia Categories)	Original AMI %	ACS Index	ACS n/N	ACS Index Components	Biomarker Criteria	Data Source	Timeframe	Notes
Räsänen et al. 1985 [36]	40	MI as underlying cause of ACPE: 19/40	47.5%	47.5%	19/40	AMI (19) as etiology of ACPE	Not specified (1985)	Weng 2010 (author data)	0–72 h	MI preceded ACPE as etiology. 9/20 NIV, 10/20 control.
Bersten et al. 1991 [19]	39	AMI: 7/39 (17.9%); Myocardial ischemia: 10/39 (25.6%)	17.9% (AMI only)	43.6%	17/39	AMI (7) + Myocardial ischemia (10)	Peak serum CK; Transmural MI: new Q-waves + CK ≥2× ULN; Nontransmural MI: ST/T changes + CK elevation	Original paper Table 1	0–72 h	Three mutually exclusive diagnoses at entry: CHF (22), Myocardial ischemia (10), AMI (7). ACS Index = sum of both ischemic categories. No reclassification performed.
Lin et al. 1995 [37]	100	AMI at study entry: 21/100	21.0%	21.0%	21/100	AMI (21)	Not specified	Original paper Table 1	0–72 h	10/50 NIV, 11/50 control.
Takeda et al. 1997 [38]	30	Acute MI as cause of ACPE: 11/30	36.7%	36.7%	11/30	Acute MI (11) — excludes "prior MI" (separate category)	Chest pain ≥30 min + CK ≥2× ULN + ECG changes	Original paper Table 1	0–72 h	5/15 NIV, 6/15 control. Prior MI listed separately.
Takeda et al. 1998 [39]	22	100% AMI population (inclusion criterion)	100%	100%	22/22	AMI (22) — inclusion criterion	Chest pain ≥30 min + CK ≥2× ULN + ECG changes	Original paper	0–72 h	Only RCT enrolling exclusively AMI patients.
Masip et al. 2000 [20]	37	AMI: 11/37 (29.7%); Unstable angina: 1/37 (2.7%)	29.7% (AMI only)	32.4%	12/37	AMI (11) + Unstable angina (1)	CK-MB (troponin not reported)	Original paper Table 2	0–72 h	AMI requiring thrombolysis excluded. UA explicitly reported.
Levitt 2001 [42]	38	AMI within 0–24 h: 9/38	23.7%	23.7%	9/38	AMI (9) — UA not reported	WHO criteria (CK-based)	Original paper	0–72 h	4/21 NIV, 5/17 control. 0–24 h timeframe.
Park et al. 2001 [21]	26	AMI: 4/26 (15.4%); Acute ischemic heart disease: 5/26 (19.2%)	15.4% (AMI only)	34.6%	9/26	AMI (4) + Acute ischemic heart disease (5)	Not specified	Original paper Table 1	0–72 h	3-arm study (O_2/CPAP/BiPAP). Etiology categories from Table 1.
Nava et al. 2003 [34]	130	AMI: 22/130	16.9%	16.9%	22/130	AMI (22)	CK-MB (enzyme protocol 0–10 h)	Winck 2006 (author data)	0–72 h	11/65 NIV, 11/65 control. Author-provided data.
Crane et al. 2004 [40]	60	AMI within 3 days: 18/60	30.0%	30.0%	18/60	AMI (18) — explicitly defined outcome	ECG changes OR CK >2× ULN (>280	Original paper	0–72 h	3-arm study. O_2 6/20, CPAP 3/20, BiPAP 9/20.

Study	N	Original Reported Data (AMI/Ischemia Categories)	Original AMI %	ACS Index	ACS n/N	ACS Index Components	Biomarker Criteria	Data Source	Timeframe	Notes
							IU/L) within 3 days			
L'Her et al. 2004 [35]	89	Acute ischemic heart disease as etiology: 13/89	14.6%	14.6%	13/89	Acute IHD (13) as etiology at baseline	Not specified	Original paper	0–72 h	7/43 NIV, 6/46 control. Lowest ACS prevalence in the dataset.
Park et al. 2004 [22]	80	NSTEMI: 5/80 (6.3%); Myocardial ischemia: 30/80 (37.5%)	6.3% (NSTEMI only)	43.75%	35/80	NSTEMI (5) + Myocardial ischemia (30)	Troponin (NSTEMI); ECG + clinical (ischemia)	Original paper Table 1	0–72 h	3-arm study. STEMI excluded by protocol. IHD total = NSTEMI + acute ischemia.
Gray et al. 2008 [13]	1069	WHO 1971 MI: ~27%; ESC/ACC 2000: ~51.5%; Symptoms at presentation: ~22%	27% (WHO 1971)	27.0%	~289/1069	MI by WHO 1971 criteria (adjudicated by 2 blinded cardiologists)	Dual: CK-MB (WHO 1971) and Troponin (ESC/ACC 2000)	Original paper; HTA monograph Tables 8 & 10	0–72 h	3CPO trial. WHO definition selected for consistency with other trials. STEMI requiring primary PCI excluded. ESC/ACC definition yields 51.5%.
Ducros et al. 2011 [41]	207	No-STe-ACS (NSTEMI/UA) at baseline: 36/207	17.4%	17.4%	36/207	NSTEMI + UA combined (not separable)	Troponin + ECG	Original paper Table 1	0–72 h	Prehospital CPAP study. STEMI excluded per protocol. 19/107 NIV, 17/100 control.

The ACS Index represents the proportion of patients with acute coronary syndrome (acute myocardial infarction and/or acute myocardial ischemia/unstable angina) documented within 0–72 hours of presentation. Data were extracted from the original trial publications using the diagnostic categories reported by each study. The Fourth Universal Definition of Myocardial Infarction [43], was used as a conceptual framework to define the temporal window (0–72 h) and the scope of acute ischemic events, not for retrospective patient-level reclassification. Where studies reported AMI and acute ischemia as separate diagnostic categories (Bersten 1991, Masip 2000, Park 2001, Park 2004), both were summed to derive the ACS Index

Abbreviations: ACS, acute coronary syndrome; ACPE, acute cardiogenic pulmonary edema; AMI, acute myocardial infarction; BiPAP, bilevel positive airway pressure; CK, creatine kinase; CK-MB, creatine kinase myocardial band; CPAP, continuous positive airway pressure; ECG, electrocardiogram; ESC/ACC, European Society of Cardiology/American College of Cardiology; HTA, Health Technology Assessment; IHD, ischemic heart disease; NSTEMI, non-ST-elevation myocardial infarction; STEMI, ST-elevation myocardial infarction; UA, unstable angina; ULN, upper limit of normal; WHO, World Health Organization.

Notes: For four studies (Bersten 1991, Masip 2000, Park 2001, Park 2004), the ACS Index exceeds the AMI-only percentage because it includes additional acute ischemic categories (myocardial ischemia, unstable angina, or acute ischemic heart disease) that were reported as separate diagnoses in the original publications. These categories represent acute coronary syndromes under contemporary classification. For Gray et al. 2008 (3CPO), the WHO 1971 definition (27%) was selected over the ESC/ACC 2000 universal definition (51.5%) to be consistent with the CK-based diagnostic criteria used in the majority of included trials.

Table S8. Meta-Regression Analysis: ACS Prevalence as Effect Modifier for NIV on Hospital Mortality.

**Panel A: Model Parameters**				
**Parameter**	**Estimate**	**SE**	**95% CI**	**p**
β_1 (ACS slope, per 1%)	−0.023	0.007	−0.042 to −0.003	0.008
R^2 (variance explained)	46.2%	—	—	—
Equilibrium point	14.1%	—	5.2%–23.0%	—
Q residual (df = 12)	4.71	—	—	0.94

**Panel B: Predicted Relative Risk at Clinical ACS Thresholds**			
**ACS Prevalence**	**Predicted RR**	**95% CI**	**Clinical Interpretation**
10%	1.10	0.72–1.67	No significant effect (RR > 1.0; 95% CI spans null)
14.1% *(equilibrium)*	1.00	—	*No effect threshold*
20%	0.87	0.66–1.15	Modest benefit (NS)
27% *(3CPO)*	0.75	0.58–0.97	Significant 25% RRR
50%	0.46	0.26–0.82	Substantial 54% RRR
100%	0.15	0.04–0.52	Maximum 85% RRR

**Panel C: Influence Diagnostics**	
**Diagnostic**	**Result**
Influential observation (Cook's D > 0.29)	[21] Park 2001: Cook's D = 0.31
High leverage point (leverage > 0.29)	[39] Takeda 1998: leverage = 0.32
Sensitivity (excluding influential cases)	β_1= −0.021 (p = 0.012); Equilibrium = 15.3%

Bold values indicate statistical significance (p < 0.05 or 95% CI excludes 1.0). Model: log(RR) = $\beta_0 + \beta_1$ × ACS prevalence.
The equilibrium point represents the ACS prevalence at which predicted RR = 1.0. At this threshold, the predicted RR equals 1.00 by mathematical definition (regression line crosses log[RR] = 0); therefore, no confidence interval for the predicted RR applies at this exact value (denoted —). The 95% CI for the equilibrium ACS prevalence threshold itself is 5.2–23.0% (see Panel A, delta method).
Abbreviations: ACS, acute coronary syndrome; CI, confidence interval; RR, relative risk; RRR, relative risk reduction; SE, standard error.

Table S9. Sensitivity and Subgroup Analyses for Hospital Mortality.

Analysis	k	N	RR (95% CI)	I^2	p-value	p-diff
**Sensitivity Analyses**						
Excluding Gray 2008 (3CPO) [13]	13	898	0.62 (0.42–0.90)	0%	0.012	—
Excluding zero-event studies	12	1904	0.74 (0.57–0.96)	0%	0.024	—
Fixed-effect model	14	1967	0.75 (0.58–0.96)	0%	0.022	—
REML estimator	14	1967	0.75 (0.58–0.96)	0%	0.022	—
Subgroup Analyses						
ACS prevalence >25%	9	1403	0.65 (0.47–0.91)	0%		
ACS prevalence ≤25%	5	564	0.93 (0.61–1.43)	0%		*0.089*
Low risk of bias only	4	1327	0.88 (0.64–1.22)	0%		
High risk of bias only	10	640	0.54 (0.35–0.83)	0%		*0.072*
CPAP only	7	527	0.68 (0.43–1.07)	0%		
BiPAP or mixed	7	1440	0.79 (0.58–1.08)	0%		*0.58*
Emergency department	8	1699	0.80 (0.61–1.06)	0%		
ICU/CCU	6	268	0.56 (0.32–0.97)	0%		*0.22*

Bold values indicate statistical significance (p < 0.05 or 95% CI excludes 1.0). p-diff: p-value for test of subgroup differences. — indicates not applicable.

Abbreviations: ACS, acute coronary syndrome; BiPAP, bilevel positive airway pressure; CCU, coronary care unit; CI, confidence interval; CPAP, continuous positive airway pressure; ICU, intensive care unit; k, number of studies; N, total participants; REML, restricted maximum likelihood; RR, risk ratio.

Table S10. Comparison of Current Meta-Analysis with Prior Meta-Analyses of NIV in ACPE.

Characteristic	**Winck 2006** [7]	**Weng 2010** [14]	**Berbenetz 2019** [9]	This Study
**Study Characteristics**				
Journal	Crit Care	Ann Intern Med	Cochrane	—
Search date	May 2005	Dec 2009	Sep 2018	Dec 2025

RCTs included (k)	17	31	24	14
Participants (N)	938	2887	2664	1967
**Inclusion Criteria**				
O_2-controlled comparator	No	No	No	Yes
ACS data required	No	No	No	Yes
Mortality data required	No	Yes	No	Yes
**Pooled Outcomes (NIV vs Standard O_2)**				
Mortality RR (95% CI)	0.47 (NR) [a]	0.64 (0.44–0.92)	0.65 (0.51–0.82)	0.75 (0.58–0.96)
Intubation RR (95% CI)	0.33 (NR) [a]	0.44 (0.32–0.60)	0.49 (0.38–0.62)	0.49 (0.35–0.68)
I^2 (mortality)	44%	NR	6%	0%
**Effect Modification Analysis**				
Meta-regression performed	No	Yes [b]	No	Yes
Effect modifier	—	MI/ischemia [b]	—	ACS (strict) [c]
Definition	—	Imprecise [b]	—	4th UD of MI [c]
Effect size (per 10% ACS)	—	17.4% RRR	—	21% RRR
R^2 (variance explained)	—	NR	—	46.2%
Equilibrium point	—	NR	—	14.1% ACS
Explains 3CPO null result	N/A	Partial	No	Yes [d]

[a] *Winck 2006 [7] reported absolute risk reduction; RR calculated from raw event data. 95% CI not reported.*

[b] *Weng 2010 [14] used "MI or ischemia at admission" (17.4% RRR per 10% increase); an imprecise definition may conflate ACS with chronic CAD.*

[c] *Present study uses strict ACS criteria based on the Fourth Universal Definition of MI (2018) [43]: STEMI/NSTEMI/UA within 0–72 hours.*

[d] *Gray 2008 (3CPO) [13] had 27% ACS prevalence but dominated the pooled analysis (n = 1069, 54.4% weight); the equilibrium point of 14.1% explains the attenuated benefit.*

Abbreviations: *3CPO, 3 Interventions in Cardiogenic Pulmonary Oedema trial; ACS, acute coronary syndrome; CAD, coronary artery disease; CI, confidence interval; I^2, heterogeneity statistic; MI, myocardial infarction; NIV, noninvasive ventilation; NR, not reported; RCT, randomized controlled trial; RR, risk ratio; RRR, relative risk reduction; UD, Universal Definition.*

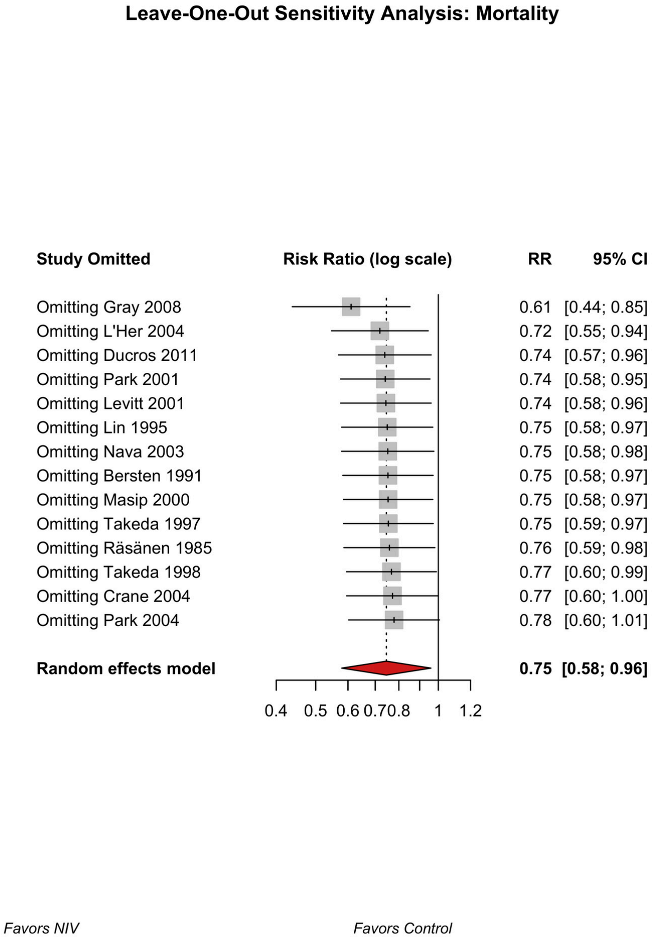

Figure S1. Leave-one-out sensitivity analysis forest plot comparing non-invasive ventilation versus standard oxygen therapy for hospital mortality in patients with acute cardiogenic pulmonary edema [13,19–22,34–42]. Each row displays the pooled effect estimate after sequentially removing the corresponding study. Squares represent the risk ratio for each iteration, with horizontal lines indicating 95% confidence intervals. The diamond represents the pooled risk ratio with its 95% confidence interval. The solid vertical line indicates no effect (RR = 1.0). RR, risk ratio; CI, confidence interval; NIV, non-invasive ventilation.

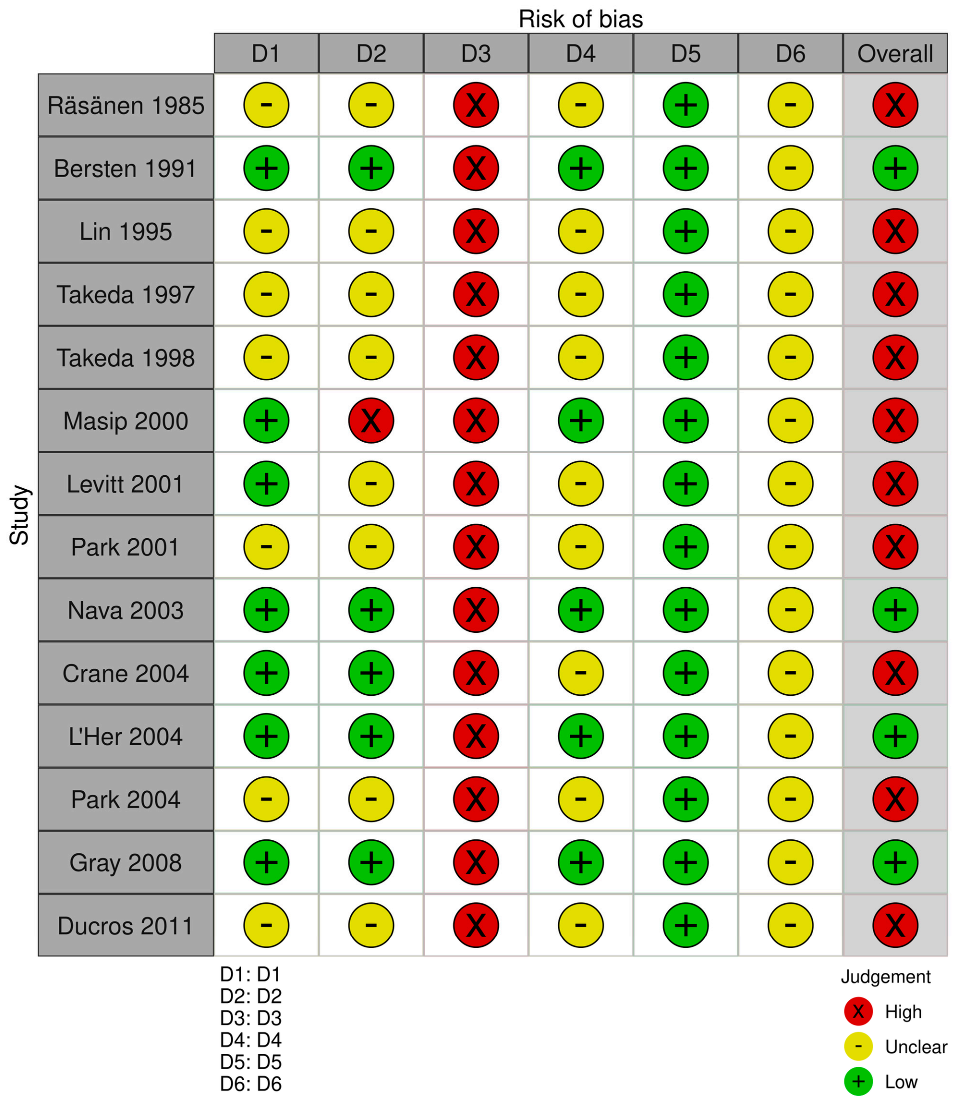

Figure S2. Risk of bias summary plot for included randomized controlled trials [13,19–22,34–42]. Bars represent the proportion of studies judged as low risk, unclear risk, or high risk across seven domains of the Cochrane Risk of Bias tool version 1 (RoB 1) [18]: (D1) random sequence generation; (D2) allocation concealment; (D3) blinding of participants and personnel; (D4) blinding of outcome assessment; (D5) incomplete outcome data; (D6) selective reporting; and (D7) other bias. Generated using the robvis package in R [24]. RoB 1, Cochrane Risk of Bias tool version 1.

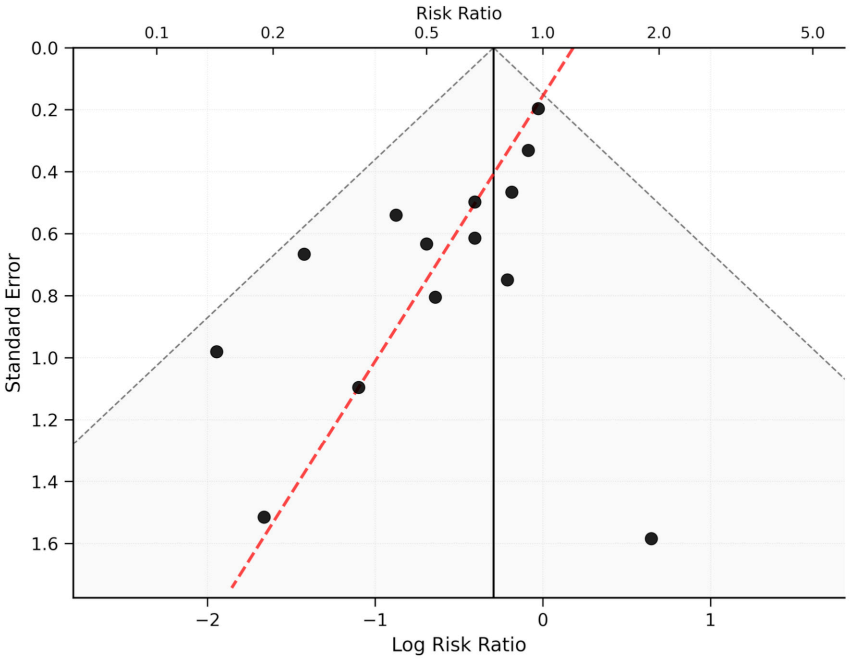

Figure S3. Contour-enhanced funnel plot of effect size (log risk ratio) against standard error for hospital mortality [13,19–22,34–42]. Each circle represents an individual study. The vertical dashed line indicates the pooled effect estimate (RR 0.75). Contour lines demarcate regions of statistical significance ($p < 0.01$, $p < 0.05$, $p < 0.10$). Funnel plot asymmetry was assessed using Egger's weighted regression test [26]; significant asymmetry was detected (intercept = −1.17, SE = 0.38, p = 0.007), suggesting potential small-study effects favoring NIV. RR, risk ratio; SE, standard error; NIV, non-invasive ventilation.

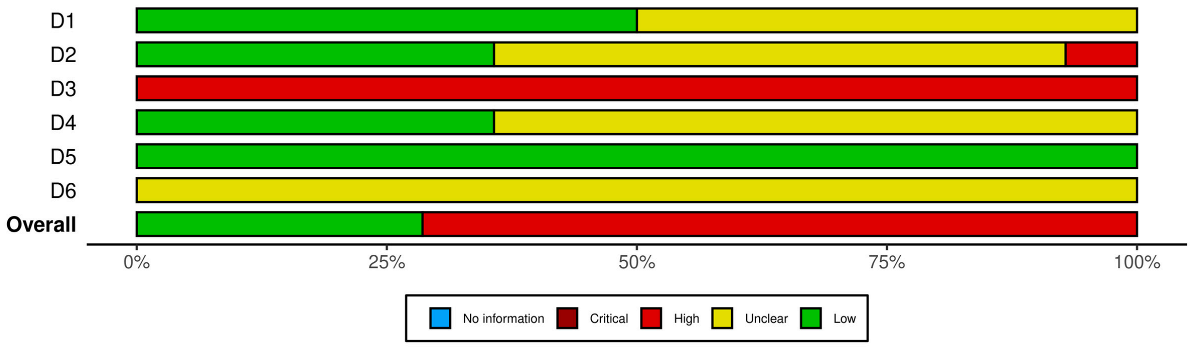

Figure S4. Risk of bias traffic-light plot for included randomized controlled trials [13,19–22,34–42], assessed using the Cochrane Risk of Bias tool version 1 (RoB 1) [18]. Each cell indicates the risk of bias judgment for an individual study across seven domains: (D1) random sequence generation; (D2) allocation concealment; (D3) blinding of participants and personnel; (D4) blinding of outcome assessment; (D5) incomplete outcome data; (D6) selective reporting; and (D7) other bias. Green = low risk; yellow = unclear risk; red = high risk. Generated using the robvis package in R [24].

References

The following references are cited in this Supplementary file. References [7], [9], [13], [14], [18]–[22], [24], [26], [30]–[35], [37]–[43] are included in the complete reference list of the main manuscript (references [1]–[55]). References [56]–[75] are cited only in the Supplementary Materials and are listed here in accordance with MDPI Supplementary Materials guidelines.

A. References Shared with Main Manuscript

7. Winck, J.C.; Azevedo, L.F.; Costa-Pereira, A.; Antonelli, M.; Wyatt, J.C. Efficacy and Safety of Non-Invasive Ventilation in the Treatment of Acute Cardiogenic Pulmonary Edema—A Systematic Review and Meta-Analysis. Crit. Care 2006, 10, R69, doi:10.1186/cc4905.
9. Berbenetz, N.; Wang, Y.; Brown, J.; Godfrey, C.; Ahmad, M.; Vital, F.M.; Lambiase, P.; Banerjee, A.; Bakhai, A.; Chong, M. Non-Invasive Positive Pressure Ventilation (CPAP or Bilevel NPPV) for Cardiogenic Pulmonary Oedema. Cochrane Database Syst. Rev. 2019, 4, CD005351, doi:10.1002/14651858.CD005351.pub4.
13. Gray, A.; Masson, M.; Sampson, F.; Nicholl, J. Noninvasive Ventilation in Acute Cardiogenic Pulmonary Edema. N. Engl. J. Med. 2008, 359, 142–151, doi:10.1056/NEJMoa0707992.
14. Weng, C.-L.; Zhao, Y.-T.; Liu, Q.-H.; Fu, C.-J.; Sun, F.; Ma, Y.-L.; Chen, Y.-W.; He, Q.-Y. Meta-Analysis: Noninvasive Ventilation in Acute Cardiogenic Pulmonary Edema. Ann. Intern. Med. 2010, 152, 590–600, doi:10.7326/0003-4819-152-9-201005040-00009.
18. Higgins, J.P.T. Measuring Inconsistency in Meta-Analyses. BMJ 2003, 327, 557–560, doi:10.1136/bmj.327.7414.557.
19. Bersten, A.D.; Holt, A.W.; Vedig, A.E.; Skowronski, G.A.; Baggoley, C.J. Treatment of Severe Cardiogenic Pulmonary Edema with Continuous Positive Airway Pressure Delivered by Face Mask. N. Engl. J. Med. 1991, 325, 1825–1830, doi:10.1056/NEJM199112263252601.
20. Masip, J.; Betbesé, A.J.; Páez, J.; Vecilla, F.; Cañizares, R.; Padró, J.; Paz, M.A.; Otero, J.D.; Ballús, J. Non-Invasive Pressure Support Ventilation versus Conventional Oxygen Therapy in Acute Cardiogenic Pulmonary Oedema: A Randomised Trial. Lancet 2000, 356, 2126–2132, doi:10.1016/S0140-6736(00)03492-9.
21. Park, M.; Lorenzi-Filho, G.; Feltrim, M.I.; Viecili, P.R.N.; Sangean, M.C.; Volpe, M.; Leite, P.F.; Mansur, A.J. Oxygen Therapy, Continuous Positive Airway Pressure, or Noninvasive Bilevel Positive Pressure Ventilation in the Treatment of Acute Cardiogenic Pulmonary Edema. Arq. Bras. Cardiol. 2001, 76, 221–230, doi:10.1590/S0066-782X2001000300005.
22. Park, M.; Sangean, M.C.; Volpe, M.D.S.; Feltrim, M.I.Z.; Nozawa, E.; Leite, P.F.; Passos Amato, M.B.; Lorenzi-Filho, G. Randomized, Prospective Trial of Oxygen, Continuous Positive Airway Pressure, and Bilevel Positive Airway Pressure by Face Mask in Acute Cardiogenic Pulmonary Edema. Crit. Care Med. 2004, 32, 2407–2415, doi:10.1097/01.CCM.0000147770.20400.10.
24. McGuinness, L.A.; Higgins, J.P.T. Risk-of-Bias VISualization (Robvis): An R Package and Shiny Web App for Visualizing Risk-of-Bias Assessments. Res. Synth. Methods 2021, 12, 55–61, doi:10.1002/jrsm.1411.
26. Egger, M.; Smith, G.D.; Schneider, M.; Minder, C. Bias in Meta-Analysis Detected by a Simple, Graphical Test. BMJ 1997, 315, 629–634, doi:10.1136/bmj.315.7109.629.
30. Plaisance, P.; Pirracchio, R.; Berton, C.; Vicaut, E.; Payen, D. A Randomized Study of Out-of-Hospital Continuous Positive Airway Pressure for Acute Cardiogenic Pulmonary Oedema: Physiological and Clinical Effects. Eur. Heart J. 2007, 28, 2895–2901, doi:10.1093/eurheartj/ehm502.
31. Moritz, F.; Brousse, B.; Gellée, B.; Chajara, A.; L'Her, E.; Hellot, M.-F.; Bénichou, J. Continuous Positive Airway Pressure Versus Bilevel Noninvasive Ventilation in Acute Cardiogenic Pulmonary Edema: A Randomized Multicenter Trial. Ann. Emerg. Med. 2007, 50, 666–675.e1, doi:10.1016/j.annemergmed.2007.06.488.
32. Frontin, P.; Bounes, V.; Houzé-Cerfon, C.H.; Charpentier, S.; Houzé-Cerfon, V.; Ducassé, J.L. Continuous Positive Airway Pressure for Cardiogenic Pulmonary Edema: A Randomized Study. Am. J. Emerg. Med. 2011, 29, 775–781, doi:10.1016/j.ajem.2010.03.007.
33. Kelly, C. Randomised Controlled Trial of Continuous Positive Airway Pressure and Standard Oxygen Therapy in Acute Pulmonary Oedema: Effects on Plasma Brain Natriuretic Peptide Concentrations. Eur. Heart J. 2002, 23, 1379–1386, doi:10.1053/euhj.2001.3156.
34. Nava, S.; Carbone, G.; DiBattista, N.; Bellone, A.; Baiardi, P.; Cosentini, R.; Marenco, M.; Giostra, F.; Borasi, G.; Groff, P. Noninvasive Ventilation in Cardiogenic Pulmonary Edema: A Multicenter Randomized Trial. Am. J. Respir. Crit. Care Med. 2003, 168, 1432–1437, doi:10.1164/rccm.200211-1270OC.
35. L'Her, E.; Duquesne, F.; Girou, E.; De Rosiere, X.D.; Conte, P.L.; Renault, S.; Allamy, J.-P.; Boles, J.-M. Noninvasive Continuous Positive Airway Pressure in Elderly Cardiogenic Pulmonary Edema Patients. Intensive Care Med. 2004, 30, 882–888, doi:10.1007/s00134-004-2183-y.
36. Räsänen, J.; Heikkilä, J.; Downs, J.; Nikki, P.; Väisänen, I.; Viitanen, A. Continuous Positive Airway Pressure by Face Mask in Acute Cardiogenic Pulmonary Edema. Am. J. Cardiol. 1985, 55, 296–300, doi:10.1016/0002-9149(85)90364-9.

37. Lin, M.; Yang, Y.-F.; Chiang, H.-T.; Chang, M.-S.; Chiang, B.N.; Cheitlin, M.D. Reappraisal of Continuous Positive Airway Pressure Therapy in Acute Cardiogenic Pulmonary Edema. Chest 1995, 107, 1379–1386, doi:10.1378/chest.107.5.1379.

38. Takeda, S.; Takano, T. The Effect of Nasal Continuous Positive Airway Pressure on Plasma Endothelin-1 Concentrations in Patients with Severe Cardiogenic Pulmonary Edema. Anesth. Analg. 1997, 84, 1091–1096, doi:10.1097/00000539-199705000-00025.

39. Takeda, S.; Nejima, J.; Takano, T.; Nakanishi, K.; Takayama, M.; Sakamoto, A.; Ogawa, R. Effect of Nasal Continuous Positive Airway Pressure on Pulmonary Edema Complicating Acute Myocardial Infarction. Jpn. Circ. J. 1998, 62, 553–558, doi:10.1253/jcj.62.553.

40. Crane, S.D. Randomised Controlled Comparison of Continuous Positive Airways Pressure, Bilevel Non-Invasive Ventilation, and Standard Treatment in Emergency Department Patients with Acute Cardiogenic Pulmonary Oedema. Emerg. Med. J. 2004, 21, 155–161, doi:10.1136/emj.2003.005413.

41. Ducros, L.; Vicaut, E.; Henry, P.; Plaisance, P.; Collet, J.-P.; Broche, C.; Gueye, P.; Vergne, M.; Goetgheber, D.; Pennec, P.-Y.; et al. CPAP for Acute Cardiogenic Pulmonary Oedema from Out-of-Hospital to Cardiac Intensive Care Unit: A Randomised Multicentre Study. Intensive Care Med. 2011, 37, 1501–1509, doi:10.1007/s00134-011-2311-4.

42. Levitt, M.A. A Prospective, Randomized Trial of BiPAP in Severe Acute Congestive Heart Failure. J. Emerg. Med. 2001, 21, 363–369, doi:10.1016/S0736-4679(01)00385-7.

43. Thygesen, K.; Alpert, J.S.; Jaffe, A.S.; Chaitman, B.R.; Bax, J.J.; Morrow, D.A.; White, H.D.; ESC Scientific Document Group. Fourth Universal Definition of Myocardial Infarction (2018). Eur. Heart J. 2019, 40, 237–269, doi:10.1093/eurheartj/ehy462.

B. References Cited Only in Supplementary Materials

56. Mehta, S.; Jay, G.D.; Woolard, R.H.; Hipona, R.A.; Connolly, E.M.; Cimini, D.M.; Drinkwine, J.H.; Hill, N.S. Randomized, Prospective Trial of Bilevel versus Continuous Positive Airway Pressure in Acute Pulmonary Edema. Crit. Care Med. 1997, 25, 620–628, doi:10.1097/00003246-199704000-00011.

57. Martin-Bermudez, R.; Babiano-Alvarez, M.A.; Bravo-Minaya, M.; de Vera-Florez, V.M. Ventilación no invasiva frente a CPAP en edema agudo de pulmón [Noninvasive Ventilation vs. CPAP in Acute Pulmonary Edema]. Med. Intensiva 2002, 26 (Suppl. 1), 135. (Spanish; abstract only; full text not retrieved)

58. Cross, A.M.; Cameron, P.; Kierce, M.; Ragg, M.; Kelly, A.M. Non-Invasive Ventilation in Acute Respiratory Failure: A Randomised Comparison of Continuous Positive Airway Pressure and Bi-Level Positive Airway Pressure. Emerg. Med. J. 2003, 20, 531–534, doi:10.1136/emj.20.6.531.

59. Liesching, T.; Nelson, D.L.; Cormier, K.L.; Sucov, A.; Short, K.; Warburton, R.; Hill, N.S. Randomized Trial of Bilevel Positive Airway Pressure vs. Continuous Positive Airway Pressure in Acute Pulmonary Edema. Am. J. Respir. Crit. Care Med. 2003, 167, A735. (Abstract only; proceedings of ATS 2003 Annual Meeting)

60. Bellone, A.; Monari, A.; Cortellaro, F.; Vettorello, M.; Arlati, S.; Coen, D. Myocardial Infarction Rate in Acute Pulmonary Edema: Noninvasive Pressure Support Ventilation Versus Continuous Positive Airway Pressure. Crit. Care Med. 2004, 32, 1860–1865, doi:10.1097/01.ccm.0000139694.47326.b6.

61. Bellone, A.; Vettorello, M.; Monari, A.; Cortellaro, F.; Coen, D. Noninvasive Pressure Support Ventilation vs. Continuous Positive Airway Pressure in Acute Hypercapnic Pulmonary Edema. Intensive Care Med. 2005, 31, 807–811, doi:10.1007/s00134-005-2649-6.

62. Ferrari, G.; Olliveri, F.; De Filippi, G.; Milan, A.; Aprà, F.; Boccuzzi, A.; Converso, M.; Navalesi, P. Noninvasive Positive Airway Pressure and Risk of Myocardial Infarction in Acute Cardiogenic Pulmonary Edema: Continuous Positive Airway Pressure vs Noninvasive Positive Pressure Ventilation. Chest 2007, 132, 1804–1809, doi:10.1378/chest.07-1058.

63. Ferrari, G.; Milan, A.; Groff, P.; Pagnozzi, F.; Mazzone, M.; Molino, P.; Aprà, F. Continuous Positive Airway Pressure vs. Pressure Support Ventilation in Acute Cardiogenic Pulmonary Edema: A Randomized Trial. J. Emerg. Med. 2010, 39, 676–684, doi:10.1016/j.jemermed.2009.07.042.

64. Fontanella, L.; Russo, M.; Viviani, M.; Trentin, L. Non-Invasive Positive Pressure Ventilation in Acute Cardiogenic Pulmonary Edema: A Randomized Multicenter Trial. Intensive Care Med. 2010, 36 (Suppl. 2), S380. (Abstract only; full text not retrievable)

65. Liesching, T.; Marnelli, A.; Cormier, K.; Nelson, D.; Short, K.; Warburton, R.; Hill, N.S. Randomized Controlled Comparison of Bilevel vs Continuous Positive Airway Pressure in Acute Cardiogenic Pulmonary Edema. Crit. Care Med. 2014, 42 (Suppl. 1), A1463. (Abstract only)

66. Rusterholtz, T.; Kempf, J.; Berton, C.; Gayol, S.; Tournoud, C.; Zaehringer, M.; Jaeger, A.; Sauder, P. Noninvasive Pressure Support Ventilation (NIPSV) with Face Mask in Patients with Acute Cardiogenic Pulmonary Edema (ACPE). Intensive Care Med. 1999, 25, 21–28, doi:10.1007/s001340050782. [Bollaert 2002 abstract presented preliminary data; this is the full publication]

67. Sharon, A.; Shpirer, I.; Kaluski, E.; Moshkovitz, Y.; Milovanov, O.; Polak, R.; Blatt, A.; Simovitz, A.; Shaham, O.; Faigenberg, Z.; et al. High-Dose Intravenous Isosorbide-Dinitrate Is Safer and Better Than Bi-PAP Ventilation Combined with Conventional Treatment for Severe Pulmonary Edema. J. Am. Coll. Cardiol. 2000, 36, 832–837, doi:10.1016/S0735-1097(00)00785-8.
68. Delclaux, C.; L'Her, E.; Alberti, C.; Mancebo, J.; Abroug, F.; Conti, G.; Guérin, C.; Schortgen, F.; Lefort, Y.; Antonelli, M.; et al. Treatment of Acute Hypoxemic Nonhypercapnic Respiratory Insufficiency with Continuous Positive Airway Pressure Delivered by a Face Mask: A Randomized Controlled Trial. JAMA 2000, 284, 2352–2360, doi:10.1001/jama.284.18.2352.
69. Ferrer, M.; Esquinas, A.; Leon, M.; Gonzalez, G.; Alarcon, A.; Torres, A. Noninvasive Ventilation in Severe Hypoxemic Respiratory Failure: A Randomized Clinical Trial. Am. J. Respir. Crit. Care Med. 2003, 168, 1438–1444, doi:10.1164/rccm.200301-072OC.
70. Thys, F.; Roeseler, J.; Reynaert, M.; Liistro, G.; Rodenstein, D.O. Noninvasive Ventilation for Acute Respiratory Failure: A Prospective Randomised Placebo-Controlled Trial. Eur. Respir. J. 2002, 20, 545–555, doi:10.1183/09031936.02.02232001.
71. El-Refay, B.H.; Ali, R.A.; Ghallab, A. Efficacy of Non-Invasive Ventilation in Management of Acute Cardiogenic Pulmonary Edema. Int. J. Adv. Res. 2016, 4, 521–529.
72. Weitz, G.; Struck, J.; Zonak, A.; Balnus, S.; Perras, B.; Dodt, C. Prehospital Noninvasive Pressure Support Ventilation for Acute Cardiogenic Pulmonary Edema. Eur. J. Emerg. Med. 2007, 14, 276–279, doi:10.1097/MEJ.0b013e32826fb377.
73. Moritz, F.; Benichou, J.; Vanheste, M.; Richard, J.-C.; Line, S.; Hellot, M.-F.; Bonmarchand, G.; L'Her, E. Boussignac Continuous Positive Airways Pressure Device in the Emergency Care of Acute Cardiogenic Pulmonary Oedema: A Randomized Pilot Study. Eur. J. Emerg. Med. 2003, 10, 204–208, doi:10.1097/00063110-200309000-00009.
74. Austin, M.A.; Wills, K.E.; Blizzard, L.; Walters, E.H.; Wood-Baker, R. Effect of High Flow Oxygen on Mortality in COPD: A Randomised Controlled Trial. BMJ 2010, 341, c5462, doi:10.1136/bmj.c5462.
75. Lin, M.; Chiang, H.T.; Chang, M.S.; Chiang, B.N.; Cheitlin, M.D. Continuous Positive Airway Pressure in Cardiogenic Pulmonary Edema (Preliminary Report). Int. J. Cardiol. 1991, 31, 209–213, doi:10.1016/0167-5273(91)90180-4. [Earlier publication from same group as Lin 1995 [37]; overlapping cohort]
76. Ferrari, G.; Groff, P.; De Filippi, G.; Giostra, F.; Mazzone, M.; Potale, G.; et al. Continuous Positive Airway Pressure (CPAP) vs. Noninvasive Positive Pressure Ventilation (NIV) in Acute Cardiogenic Pulmonary Edema (ACPE): A Prospective Randomized Multicentric Study. J. Emerg. Med. 2006, 30, 246–247.
77. Hao, C.X.; Luo, L.X.; Liu, Y.M. Treatment of severe cardiogenic pulmonary edema with continuous positive airway pressure by nasal face mask. Acta Academiae Medicinae Jiangxi (Jiangxi Yixueyuan Xuebao) 2002, 42, 48–50. (Chinese-language publication; not indexed in PubMed/MEDLINE; no DOI).
78. Li, X.K.; Zhao, W.H. The treatment of acute cardiogenic pulmonary edema with noninvasive positive pressure ventilation. Chongqing Medical Journal 2005, 34(4), 575–576. (Chinese-language publication; not indexed in PubMed/MEDLINE; no DOI).
79. Zokaei, A.; Ahmadi, S.J.; Hemmati, N. Noninvasive Pressure Control Inverse Ratio Ventilation (NIPCIRV) versus conventional oxygen therapy for treatment of acute cardiogenic pulmonary edema. Research Journal of Medical Sciences 2016, 10(5), 449–452. https://doi.org/10.3923/rjmsci.2016.449.452
80. Agmy, G.; Makhlouf, H.; Mohammed, A.; Ghanem, M.; Mohammed, H. CPAP versus BiPAP in Acute Cardiogenic Pulmonary Edema: Experience with 129 Patients. In Proceedings of the European Respiratory Society Annual Congress, Berlin, Germany, 4 October 2008. (Conference abstract; full text not retrieved; data discrepancies identified with ClinicalTrials.gov registration NCT00912158)
